# Hydrodynamics of a Confined Surfactant-Laden Compound Droplet under Steady and Pulsatile Flows

**DOI:** 10.1021/acs.langmuir.6c02929

**Published:** 2026-09-23

**Authors:** Shubham Lanjewar, Priyesh Tongaria, Sundari Ramji

**Affiliations:** Department of Chemical Engineering, 29794 BITS Pilani K K Birla Goa Campus, Zuarinagar 403726, India

## Abstract

Compound droplets, consisting of a liquid core encapsulated within another liquid shell, find extensive applications in the food, pharmaceutical, and cosmetic industries to encapsulate nutrients, flavors, and active ingredients, as well as in drug delivery and the fabrication of porous particles and functional materials. These compound droplets are generally stabilized by surfactants, which increase interfacial deformability, thereby preventing coalescence. Motivated by these applications, this study numerically investigates the dynamics of a deformable compound droplet laden with an insoluble surfactant within a vertical channel. An in-house solver based on the level-set method is employed to analyze the interfacial transport under both steady and pulsatile inflows. Three initial surfactant configurations are considered: (C1) surfactant present only on the outer shell interface, (C2) surfactant distributed only on the inner core interface, and (C3) surfactant present on both interfaces. This work presents the first systematic investigation simultaneously considering multiple surfactant distribution scenarios and different inflow conditions. The study reveals the existence of relatively stronger surfactant concentration gradients on the outer shell interface, which is directly exposed to the external flow, while the inner core interface remains encapsulated and largely isolated from the effects of the imposed inflow. Consequently, configurations with surfactant on the shell (cases (C1) and (C3)) have a higher impact on droplet velocity, shell–core deformation, and core eccentricity, compared with surfactant on the core (C2), under both steady and pulsatile inflows. Moreover, our analysis of the coupled interaction between pulsation frequency and surfactant transport indicates that the Strouhal number (*St*) has a more pronounced effect on the overall droplet dynamics than the Péclet number (*Pe*).

## Graphical abstract

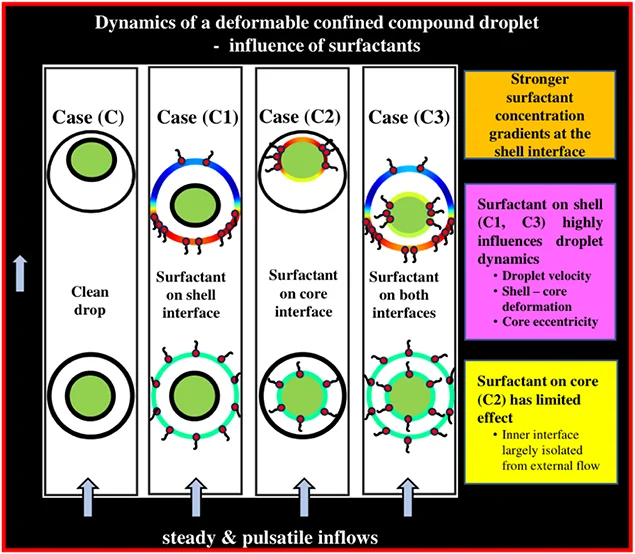

## Introduction

Surfactants play a crucial role in controlling droplet formation and stability, functions which are essential across diverse sectors including healthcare and medicine,^1^ food and pharmaceuticals,^1^ material science and catalysis and in chemical process technology.^2^ Their unique molecular structure containing both a hydrophilic and a hydrophobic part allows them to accumulate over the interfluid interface, thereby reducing the interfacial tension between immiscible fluids like oil and water. This reduction in interfacial tension renders the interface deformable, prevents coalescence and stabilizes droplets in emulsions.^3^

Although surfactants are employed in both batch (large-scale) and microfluidic processes, their role in microfluidics is particularly crucial for achieving precise control over droplet formation, size distribution, and morphology including the generation of simple and core–shell (compound) droplets with well-defined numbers of encapsulated cores. In droplet-based microfluidic systems,^3,4^ surfactants stabilize droplets that function as isolated microreactors by preventing coalescence, thereby preserving compartmentalization and preventing cross-contamination. Microfluidic droplet reactors^4,5^ further benefit from a high surface-area-to-volume ratio, which significantly enhances mass and heat transfer, leading to faster and more controlled reaction kinetics. Surfactants also regulate interfacial stresses and internal circulation within droplets, thereby influencing mixing, encapsulation efficiency, and release characteristics. As a result, surfactant-laden droplets have found widespread applications in targeted drug delivery and controlled release systems,^6^ single-cell analysis and biological assays,^7^ high-throughput screening and diagnostics,^8^ synthesis of functional and porous materials such as microspheres and capsules,^9^ food and cosmetic encapsulation technologies,^10^ and advanced material fabrication including double emulsions for templating and particle synthesis.^11^ More recently, droplet-based bioprinting and cell encapsulation technologies have been developed for regenerative medicine applications,^12^ and artificial cell and protocell synthesis, where multicompartment droplets mimic cellular architectures for studying biological processes.^13^ Therefore, a detailed understanding of surfactant dynamics at fluid interfaces, particularly in compound droplet systems, is essential for optimizing their performance across a wide range of emerging microfluidic applications.

Several studies have investigated the role of surfactants on the dynamics of both confined and unconfined simple droplet using experiments,^14–16^ theory and numerical simulations.^17–21^ Numerical simulations have been instrumental in uncovering this complex interfacial and hydrodynamic interactions. The behavior of surfactant-covered droplets in extensional flow has been widely studied using numerical and analytical approaches, revealing critical insights into droplet deformation, stability, and breakup mechanisms. One of the earliest numerical studies by Stone and Leal^22^ employed the boundary-integral method for low *Re* flow, to investigate the dynamics of a neutrally buoyant droplet covered with an insoluble surfactant. They describe two competing mechanisms that govern the dynamics of a surfactant-laden droplet subjected to an external flow. First, the interfacial flow convects surfactant toward the rear end of the droplet, resulting in a local accumulation of surfactant. This lowers the local interfacial tension and promotes greater deformation. Second, as the droplet stretches, the interfacial area increases causing the surfactant to become locally diluted, i.e., the surfactant concentration decreases. This dilution increases the local interfacial tension and opposes further deformation. They reported that surfactants increase deformation at low capillary numbers by lowering interfacial tension, but at higher capillary numbers, surfactant dilution counteracts this effect, altering the critical breakup conditions. Other researchers^23,24^ extended this work by exploring the role of surfactant solubility on the deformation and breakup of droplets, demonstrating that insoluble surfactants generate Marangoni stresses that slow interfacial motion, enhance early time deformation, and delay breakup, while the presence of soluble surfactants reduce interfacial tension gradients through adsorption–desorption exchange, thereby weakening Marangoni effects and diminishing the overall drop stabilization. Specifically, all these studies employed linear equation of state to relate surface tension to the surfactant concentration. A more detailed investigation of surfactant transport kinetics employing the nonlinear equation of state was reported by several researchers,^25–27^ who examined how elasticity and initial surfactant coverage influence tip streaming and breakup behavior during extensional flow. Kruijt-Stegeman et al.^28^ used finite element method to analyze droplet elongation and breakup with insoluble surfactants under negligible inertia and supercritical flow for viscosity matched systems. Recent studies^29,30^ also explore the role of surface viscosity providing crucial insights on tip streaming and stability of a drop under extensional flow. In particular, most of the above-mentioned studies dealt with droplets at low *Re* suspended in unconfined domains and mostly under neutrally buoyant conditions.

Following this, several researchers have numerically analyzed the dynamics of simple drops under confined shear flows at low^31–33^ and finite *Re*.^20,34^ Collectively, they report surfactant-covered drops to become more elongated and less tilted than clean drops, promoting breakup at lower capillary numbers with smaller daughter droplets, whereas at high inertia, Marangoni forces dominate, causing the drop to retract delaying breakup. Xu et al. in 2012 ^34^ developed a level-set based continuum surface force method for simulating two-phase flows with insoluble surfactants, enabling detailed analysis of droplet deformation, coalescence prevention, and multiple droplet interactions. Using a phase-field model within the lattice Boltzmann framework, Shi et al.^21^ studied droplets with soluble surfactants. Their results showed that increasing surfactant concentration enhances droplet deformation and leads to more complex droplet–droplet interactions.

Although numerous numerical studies have examined simple droplets under extensional and shear flows, only a limited studies have explored the effects of surfactants in uniform and Poiseuille flows. Rednikov et al.^35^ showed that surfactant transport controls droplet deformation and trajectory in uniform flows by creating interfacial tension gradients. Li and Pozrikidis^36^ demonstrated that surfactant properties, including elasticity and interfacial concentration, dictate transient shape evolution of droplets under creeping flow. Almatroushi and Borhan^17^ found that surfactants alter buoyancy-driven motion of viscous drops and bubbles, modifying rise velocity and stabilizing or destabilizing the interface in confined domains. Sman and Graaf^37^ developed a free-energy-based lattice Boltzmann model that accurately simulated surfactant adsorption at curved fluid interfaces. In microchannels, Luo et al.^18,19^ used front-tracking methods to study both insoluble and soluble surfactants in square microchannels, showing that interfacial surfactant gradients significantly influence deformation, increase pressure drop, and slow droplet movement. In pressure-driven channel flows, Ahmed et al.^38^ showed that surfactant solubility and nonuniform distribution govern bubble lateral migration, stability, and deformation in pressure-driven channel flows. Similarly, Garg et al.^39^ employed direct numerical simulations (DNS) to study the extraction of contaminants by a single droplet in yield-stress fluids, accounting for complex droplet–fluid interactions and rheological effects.

The dynamics of compound droplets have attracted considerable attention in recent years due to their relevance in microfluidic applications such as drug delivery, controlled encapsulation, and biochemical assays. In comparison to the existing studies on clean compound droplets,^40–42^ numerical investigations focusing on surfactant-laden compound droplets are still scarce. Mandal et al., 2016 ^43^ conducted a foundational theoretical analysis of compound droplets in arbitrary unbounded Stokes flows, accounting for the presence of surfactants on both the core and shell interfaces. Their study demonstrated how surfactant-induced Marangoni stresses can significantly alter droplet deformation, eccentricity, and suspension rheology. Further, Wang et al.^44^ experimentally and Li et al.^45^ numerically explored the self-propulsion of compound droplets driven by interfacial Marangoni effects, uncovering a wide range of droplet trajectories depending on internal viscosity, volume ratios, and surfactant transport. More recently, a numerical model to simulate how surfactants influence deformation, merging, and breakup of multicore droplets in shear flow was developed by Duong et al.,^46^ where they identified regime transitions controlled mainly by capillary number, elasticity, and Péclet number. Despite the extensive work on simple droplets, numerical studies addressing surfactant dynamics in confined compound droplets thus remain quite limited.

Investigating compound droplet dynamics under pulsatile flow is important because many real-world systems such as blood flow, microfluidic pumps, and drug delivery devices operate under time-varying flow conditions.^47^ Pulsatile flow introduces oscillatory forces that significantly influence droplet deformation, transport, and mixing behavior, distinct from that under steady flow, making it essential for designing precise and efficient fluid-based systems. Most of the earlier studies have focused on the dynamics of a compound droplet under steady or unidirectional flows in the absence of surfactants. Our earlier works on the dynamics of a compound droplet/bubble under an imposed pulsatile flow^48,49^ systematically investigated how a clean compound droplet/bubble exhibit shape oscillations and eccentric migration due to the cyclic nature of the imposed flow. The present study extends this work by incorporating insoluble surfactant on the core, shell, and both interfaces of the compound droplet, thereby addressing the scenario of surfactant-laden compound droplets under steady and pulsatile flows, accounting for the combined effects of inertia, viscosity, pressure, gravity, capillary, and Marangoni forces. The present study provides new insights into the evolution of different initial surfactant distributions on the shell and core interfaces and their consequent effects on shell and core deformation, droplet velocity, core eccentricity, and the overall interfacial dynamics. It further elucidates how periodic flow reversal modifies surfactant redistribution and, consequently, the hydrodynamics of the compound droplet. In addition, the study systematically quantifies the shielding effect of the outer shell on surfactant transport and interfacial dynamics at the core interface across a range of core sizes, thereby revealing how confinement governs the behavior of the compound droplet. To our knowledge, this is the first numerical study to systematically address the combined influence of pulsatile forcing and surfactant-mediated interfacial dynamics in compound droplet, building directly on the pulsatile flow framework introduced in our previous work.^48^

This paper begins by explaining model development, followed by model validation. Further, the results are analyzed by comparing clean and surfactant-laden droplets under both steady and pulsatile inflows to establish the baseline behavior and highlight the influence of time-dependent forcing. Three surfactant loading configurations (viz. core-only, shell-only, and both interfaces) are then analyzed to reveal deformation patterns, core eccentricity, droplet speed and interfacial stress distribution.

## Model Development

### Problem Statement and System Geometry

The motion of a deformable, surfactant-laden compound droplet—comprising of a liquid shell (*s*) encapsulating a liquid core (*c*) and suspended in a continuous fluid (*o*)—is analyzed within a vertical channel as depicted in Figure 1. Initially, the compound droplet is circular and concentric with the radii of the shell and core denoted by R̃*_s_* and R̃*_c_* respectively. The channel has a width *D* and length *L*, with rigid walls. In our previous work,^48^ an in-house solver based on the level set method was developed to study the motion of a clean, surfactant-free compound droplet under pulsatile flow. Building on this, we extend the framework to incorporate surfactant effects by considering three distinct surfactant distribution scenarios under both steady and pulsatile flow—an investigation undertaken here for the first time. Accordingly, the present study examines three distinct initial surfactant distribution scenarios, as illustrated in Figure 1b–1d. In the first case (C1) (Figure 1b), surfactant is present only at the continuous fluid-shell interface (referred to as shell interface henceforth), while the shell–core interface (core interface henceforth) remains clean. In the second case (C2) (Figure 1c), the surfactant is confined to the core interface, with the shell interface left clean. In the third case (C3) (Figure 1d), both interfaces are coated with surfactant. These surfactant configurations are consistent with experimental findings reported in earlier studies.^50^

**Figure 1:**
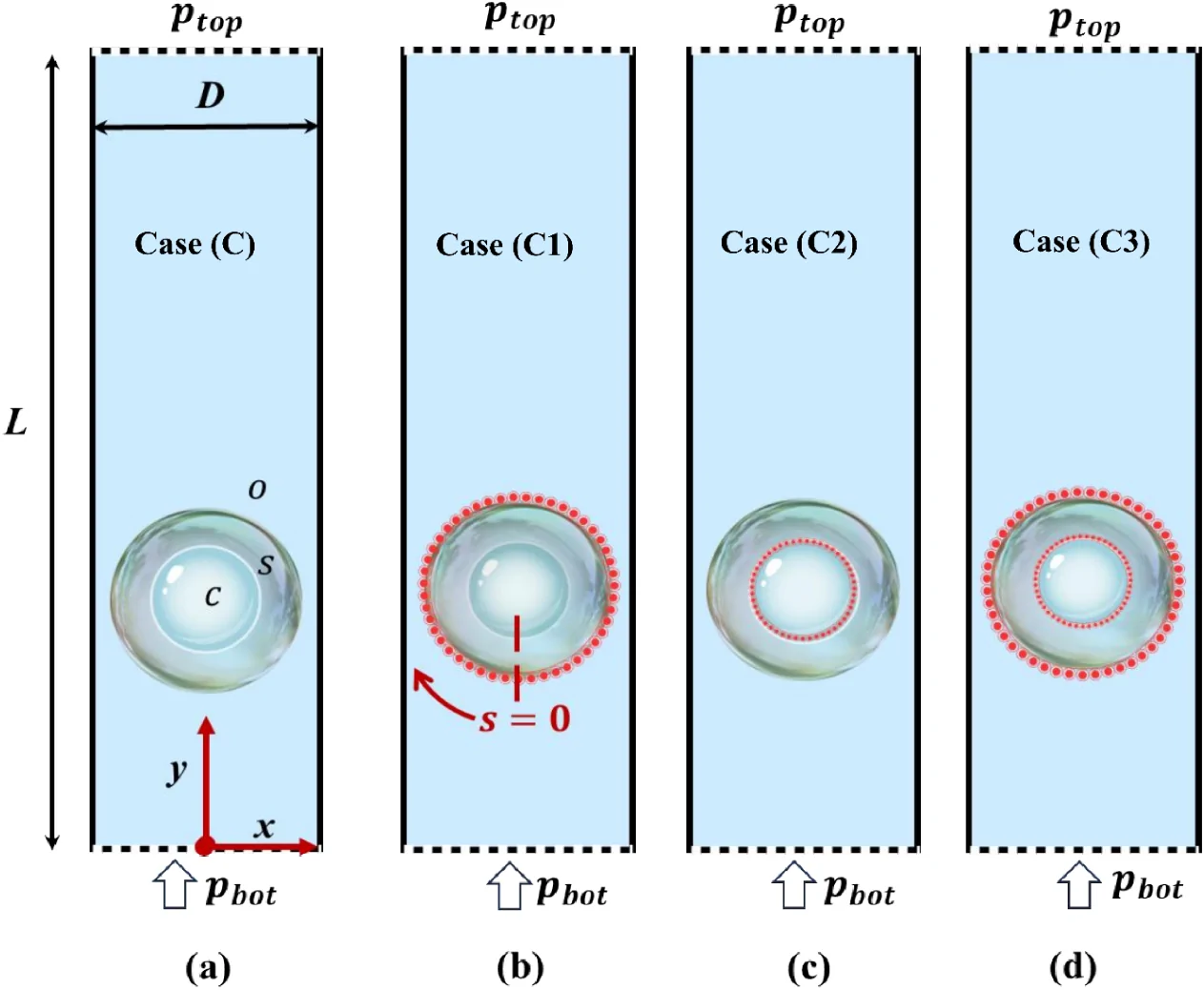
(a) Illustration of the system geometry, showing a clean compound droplet suspended in a surrounding continuous fluid. The system consists of three distinct fluid phases: the continuous phase (*o*), the shell (*s*), and the core (*c*) fluids. Panels (b), (c), and (d) depict various surfactant distribution scenarios: (C1) surfactant localized at the shell interface (Γ*os*) with a clean core, (C2) surfactant present at the core interface (Γ*sc*) with a clean shell, and (C3) surfactant distributed at both interfaces. In panel (b), *s* represents the arc length, which is zero at the rear of the shell as shown.

A two-dimensional (2D) computational model is employed in this study for its simplicity and ability to capture key physical aspects of the surfactant-laden compound droplet dynamics, while ensuring low computational cost. Previous studies based on 2D models have demonstrated strong agreement with experiments.^48,51^ Further, experimental observations show that a thin film of the continuous fluid always surrounds the compound droplet, preventing its direct contact with the channel walls^52,53^ and hence contact line is not included in our previous works^48,49^ as well as in the current model. Previous numerical studies often assumed the core to possess the same properties as the continuous fluid.^42,54–56^ However, real-world scenarios frequently involve compound droplets where the properties of the three fluids are different.^52,53^ Consequently, the three fluids—continuous phase (*o*), shell (*s*), and core (*c*)—are modeled with distinct physical properties. In the presence of three immiscible fluids, there are two interfaces: Γ*_os_*, which separates the continuous phase and shell fluid, and Γ*_sc_*, which separates the shell and core fluids. The density and viscosity of each phase are denoted as ρ̃*_i_* and μ̃*_i_*, respectively (where *i* represents phases *o*, *s* and *c*), while the interfacial tensions between the outer-shell and shell–core fluids are represented as σ̃*_os_* and σ̃*_sc_*, respectively. Here, quantities with a tilde (∼) indicate dimensional values.

Flow within the channel is driven by an imposed pressure gradient. At the channel inlet (bottom), a nondimensional positive pressure (*p_bot_* > 0) is applied in addition to the hydrostatic pressure to impart upward flow of the continuous phase. At the outlet (top), the pressure *p_top_* is set to zero. Gravity acts downward along the *y*-axis. To study the behavior of the compound droplet under pulsatile flow conditions, a periodic pressure is applied at the inlet, as described by eq 1. This periodic pressure combines an oscillating component with the mean pressure drop *p*_0_, which is equal to the sum of the hydrostatic pressure and the imposed pressure, Δ*p*.^48^

1 $$p_{\mathrm{bot}}=p_{0}\left(1+A\sin\left(2\pi t\right)\right)$$

In eq 1, *A* represents the dimensionless amplitude of the oscillation and *t*, the dimensionless time. *A* = 0 represents a steady inflow in the domain.

### Level Set Formulation for Multifluid Interface

In our previous work,^48^ we employed the level set method to simulate compound droplets accounting for distinct physical properties for each of the three fluids. The same approach is adopted here to model multifluid interfaces, with a brief overview provided below. For a detailed description, readers are referred to our earlier work.

In the presence of three fluids and two interfaces, two level set functions, *ϕ_os_* and *ϕ_sc_* are employed. The zero-contour of the level set function *ϕ_os_* represents the shell interface (i.e., *ϕ*_os_= 0 at Γ_os_), with *ϕ*_os_> 0 everywhere outside and *ϕ*_os_< 0 inside it. Similarly, the zero contour of the level set function *ϕ_sc_* represents the core interface (i.e., *ϕ*_sc_= 0 at Γ*_sc_*), with *ϕ_sc_* taking positive (negative) values in the shell and continuous (core) fluids. Thus, the level set function ϕ*_j_* (where *j* represents *os*, *sc* fluid pair) is described by eq 2 as,

2 $$\phi_{j}=\{\begin{array}{ll} +d_{j} & if(x,y)\;is\;outside\;\Gamma_{j},\\ 0 & at\;\Gamma_{j},\\ -d_{j} & if\;(x,y)\;is\;inside\;\Gamma_{j} \end{array}$$

where *d_j_* in eq 2 is the distance function, which gives the shortest distance from the interface Γ*_j_*. We further define the smoothened Heaviside function *H_j_* to represent a diffuse interface such that fluid properties vary smoothly across the phases (discussed later), as given by eq 3. The interfluid interface is smoothened to have a finite thickness occupying the region within which −ϵ ≤ *ϕ_j_* ≤ ϵ. Here, ϵ determines the thickness of the interface and is taken equal to the grid size, *dx*.

3 $$H_{j}=\{\begin{array}{ll} 0 & if\;\phi_{j}<-\epsilon ,\\ 1 & if\;\phi_{j}>\epsilon ,\\ 0.5\left(1+\frac{\phi_{j}}{\epsilon }+\frac{\sin\left(\frac{\pi \phi_{j}}{\epsilon }\right)}{\pi }\right) & otherwise \end{array}$$

The smoothened delta function δ*_j_* which is the derivative of the Heaviside function is given by eq 4.

4 $$\delta_{j}=\{\begin{array}{ll} \frac{1}{2\epsilon }\left(1+\cos\left(\frac{\pi \phi_{j}}{\epsilon }\right)\right) & if\;\left|\phi_{j}\right|\leq \epsilon ,\\ 0 & else \end{array}$$

### Governing Equations for Fluid Flow

The behavior of a compound droplet is influenced by the combined effects of external flow, capillary forces, buoyancy, and viscous forces. To accurately determine the motion of a compound droplet within a vertical channel, the dimensionless forms of the mass conservation and momentum equations are solved in a Cartesian coordinate system. The momentum equation is expressed using the single-fluid formulation developed by Brackbill et al.^57^ which incorporates the interfacial forces within the momentum equation, localizing it to the interfacial region. In this study, the fluids are assumed to be Newtonian and incompressible. As the compound droplet moves with the surrounding flow, the interfacial tensions at the shell and core interfaces (denoted by σ̃*_os_* and σ̃*_sc_*, respectively) become variable due to its dependence on surfactant concentration, resulting in the Marangoni effect—surface tension gradient-driven flow due to nonuniform surfactant concentration along the interfaces.^26,27^

The dimensionless mass conservation and momentum balance equations are described by eqs 5–7. Here, *u* and *v* denote the velocity components in the *x*- and *y*-directions, respectively, and *p* represents the pressure. The characteristic scales used for obtaining the governing equations in the dimensionless form are

$$v_{ch}=\sqrt{g{\mathrm{D̃}}_{s}},t_{ch}=\frac{2\pi }{\omega },x_{ch}={\mathrm{D̃}}_{s},\rho_{ch}={\mathrm{\rho ̃}}_{o},\mu_{ch}={\mathrm{\mu ̃}}_{o},\kappa_{ch}=\frac{1}{x_{ch}},\sigma_{ch}={\mathrm{\sigma ̃}}_{0}$$

and

$$p_{ch}=\rho_{ch}v_{ch}^{2}$$

Here, D̃*_s_* is the initial shell diameter and *ω* is the angular frequency of pulsation. Furthermore, the Strouhal number, $St=\frac{\omega }{2\pi }\sqrt{\frac{{\mathrm{D̃}}_{s}}{g}}$ ; the Archimedes number, $Ar=\frac{{\mathrm{D̃}}_{s}^{3/2}g^{1/2}{\mathrm{\rho ̃}}_{o}}{{\mathrm{\mu ̃}}_{o}}$ and Bond number, $Bo_{j}=\frac{{\mathrm{\rho ̃}}_{o}g{\mathrm{D̃}}_{s}^{2}}{{\mathrm{\sigma ̃}}_{0,j}}$ for *j* = *os*, *sc* fluid pair, where σ̃*_o,j_* represents the dimensional interfacial tension corresponding to a clean interface, and is assumed to be the same for both fluid pairs in this study.^34^ The choice of characteristic scales adopted in the present study follows several previous works,^48,58,59^ and is primarily for the sake of consistency with the literature. Importantly, all terms in the governing equations are retained during the nondimensionalization; therefore, no physical effects are ignored.

5 $$\frac{\partial u}{\partial x}+\frac{\partial v}{\partial y}=0$$

6 $$St\frac{\partial u}{\partial t}+u\frac{\partial u}{\partial x}+v\frac{\partial u}{\partial y}=-\frac{1}{\rho }\frac{\partial p}{\partial x}+\frac{1}{\rho Ar}\left[\frac{\partial }{\partial x}\left(2\mu \frac{\partial u}{\partial x}\right)+\frac{\partial }{\partial y}\left(\mu \frac{\partial v}{\partial x}\right)+\frac{\partial }{\partial y}\left(\mu \frac{\partial u}{\partial y}\right)\right]+\frac{F_{x}}{\rho }$$

7 $$St\frac{\partial v}{\partial t}+u\frac{\partial v}{\partial x}+v\frac{\partial v}{\partial y}=-\frac{1}{\rho }\frac{\partial p}{\partial y}+\frac{1}{\rho Ar}\left[\frac{\partial }{\partial y}\left(2\mu \frac{\partial v}{\partial y}\right)+\frac{\partial }{\partial x}\left(\mu \frac{\partial v}{\partial x}\right)+\frac{\partial }{\partial x}\left(\mu \frac{\partial u}{\partial y}\right)\right]+\frac{F_{y}}{\rho }-1$$

In eqs 6–7, *ρ* and *μ* represent the nondimensional density and viscosity in the entire domain. The Heaviside function defined in eq 3 ensures the fluid properties change smoothly over a few grids close to the interface. The density *ρ* and viscosity *μ* are then computed from eqs 8 and 9, respectively.

8 $$\rho =\{\begin{array}{ll} 1, & if\;\phi_{os}>\epsilon \&\phi_{sc}>\epsilon ,\\ \left(1-{\mathrm{\rho ̂}}_{s}\right)H_{os}+{\mathrm{\rho ̂}}_{s}, & if\;\left|\phi_{os}\right|\leq \epsilon ,\\ {\mathrm{\rho ̂}}_{s}, & if\;\phi_{os}<-\epsilon \&\phi_{sc}>\epsilon ,\\ \left({\mathrm{\rho ̂}}_{s}-{\mathrm{\rho ̂}}_{c}\right)H_{sc}+{\mathrm{\rho ̂}}_{c}, & if\;\left|\phi_{sc}\right|\leq \epsilon ,\\ {\mathrm{\rho ̂}}_{c}, & otherwise \end{array}$$

where,

$${\mathrm{\rho ̂}}_{c}=\frac{{\mathrm{\rho ̃}}_{c}}{{\mathrm{\rho ̃}}_{o}};{\mathrm{\rho ̂}}_{s}=\frac{{\mathrm{\rho ̃}}_{s}}{{\mathrm{\rho ̃}}_{o}}$$

Similarly,

9 $$\mu =\{\begin{array}{ll} 1, & if\;\phi_{os}>\epsilon \&\phi_{sc}>\epsilon ,\\ \left(1-{\mathrm{\mu ̂}}_{s}\right)H_{os}+{\mathrm{\mu ̂}}_{s}, & if\;\left|\phi_{os}\right|\leq \epsilon ,\\ {\mathrm{\mu ̂}}_{s}, & if\;\phi_{os}<-\epsilon \&\phi_{sc}>\epsilon ,\\ \left({\mathrm{\mu ̂}}_{s}-{\mathrm{\mu ̂}}_{c}\right)H_{sc}+{\mathrm{\mu ̂}}_{c}, & if\;\left|\phi_{sc}\right|\leq \epsilon ,\\ {\mathrm{\mu ̂}}_{c}, & otherwise \end{array}$$

where,

$${\mathrm{\mu ̂}}_{c}=\frac{{\mathrm{\mu ̃}}_{c}}{{\mathrm{\mu ̃}}_{o}};{\mathrm{\mu ̂}}_{s}=\frac{{\mathrm{\mu ̃}}_{s}}{{\mathrm{\mu ̃}}_{o}}$$

Moreover, in eqs 6–7, *F_x_* and *F_y_* are the total interfacial forces. The total interfacial forces in the absence of surfactants are entirely determined by the interface curvature and the constant interfacial tension. However, in surfactant-laden droplet flows, the presence of surfactants alters the interfacial tension locally depending on the concentration of surfactant. These variations give rise to Marangoni stress, which induce tangential flow along the interface, thereby influencing the droplet’s shape and overall dynamics.^26,60^

Thus, in the presence of surfactant, the interfacial force in each fluid pair is a combination of the capillary force (*i*.*e*., −*σ_j_k_j_ **n**_j_*) due to interface curvature, and the Marangoni force (*i*.*e*., ∇*_s_σ_j_*) due to surface tension gradients.^34^ In the *x*-direction, the interfacial forces for the *j* ^th^ fluid pair, $F_{x_{j}}$ are described by eqs 10–12.

10 $$F_{x_{j}}=F_{x_{j},Ma}+F_{x_{j},Ca}$$

Here,

11 $$\begin{array}{ll} F_{x_{j},Ma} & =\frac{\left(\nabla_{s}\sigma_{j}\delta \left(\phi_{j}\right)\left|\nabla \phi_{j}\right|\right)}{Bo_{j}}\\ & =\frac{1}{Bo_{j}}\left[\delta \left(\phi_{j}\right)\left|\nabla \phi_{j}\right|\left(\frac{\partial \sigma_{j}}{\partial x}-n_{x,j}\left(n_{x,j}\frac{\partial \sigma_{j}}{\partial x}+n_{y,j}\frac{\partial \sigma_{j}}{\partial y}\right)\right)\right] \end{array}$$

where $n_{j}=\left[n_{x,j},n_{y,j}\right]=\frac{\nabla \phi_{j}}{\left|\nabla \phi_{j}\right|}$ is the unit normal vector, and

12 $$F_{x_{j},Ca}=\frac{-\left(\sigma_{j}\kappa_{j}\nabla H\left(\phi_{j}\right)\right)}{Bo_{j}}$$

The interfacial forces in the *y*-direction are determined using the same approach. Now, the total surface force components in the *x* and *y* directions will then be $\left(F_{x_{os}}+F_{x_{sc}}\right)$ and $\left(F_{y_{os}}+F_{y_{sc}}\right)$ respectively. For the three different surfactant distribution scenarios, the total surface force in the *x*-direction is formulated, as shown in eq 13, with analogous expressions applicable in the *y*-direction.

13 $$\begin{array}{ll} C1:\mathrm{Surfactant}\;\mathrm{on}\;\mathrm{the}\;\mathrm{shell}\;\mathrm{interface}\;\mathrm{alone} & F_{x}=F_{x_{os},Ma}+F_{x_{os},Ca}+F_{x_{sc},Ca}\\ C2:\mathrm{Surfactant}\;\mathrm{on}\;\mathrm{the}\;\mathrm{core}\;\mathrm{interface}\;\mathrm{alone} & F_{x}=F_{x_{sc},Ma}+F_{x_{sc},Ca}+F_{x_{os},Ca}\\ C3:\mathrm{Surfactant}\;\mathrm{on}\;\mathrm{both}\;\mathrm{interfaces} & F_{x}=F_{x_{os},Ma}+F_{x_{os},Ca}+F_{x_{sc},Ma}+F_{x_{sc},Ca} \end{array}$$

In eq 12, *κ_j_* represents the dimensionless curvature corresponding to the *j* ^*th*^ fluid pair and is expressed as

14 $$\kappa_{j}=\frac{\left(\frac{\partial^{2}\phi_{j}}{\partial x^{2}}\right){\left(\frac{\partial \phi_{j}}{\partial y}\right)}^{2}-2\left(\frac{\partial \phi_{j}}{\partial x}\right)\left(\frac{\partial \phi_{j}}{\partial y}\right)\left(\frac{\partial^{2}\phi_{j}}{\partial x\partial y}\right)+\left(\frac{\partial^{2}\phi_{j}}{\partial y^{2}}\right){\left(\frac{\partial \phi_{j}}{\partial x}\right)}^{2}}{{\left({\left(\frac{\partial \phi_{j}}{\partial x}\right)}^{2}+{\left(\frac{\partial \phi_{j}}{\partial y}\right)}^{2}\right)}^{3/2}}$$

The dimensionless interfacial tension, σ_*j*_, in eqs 10–12, depends on the dimensionless surfactant concentration, *f_j_*. Their relationship is described by the nonlinear Langmuir equation of state,^34^ expressed as

15 $$\sigma_{j}=1+\beta \ln\left(1-\xi f_{j}\right)$$

for *j* = *os* and *sc* fluid pair.

The important parameters in eq 15 are the surfactant elasticity, $\beta =\frac{RT{\mathrm{f̃}}_{\infty ,j}}{{\mathrm{\sigma ̃}}_{o,j}}$ and the surfactant coverage, $\xi =\frac{{\mathrm{f̃}}_{0,j}}{{\mathrm{f̃}}_{\infty ,j}}$ . Notably, *β* = 0 for a clean interface. Further, f̃_0,_ *_j_* and f̃_∞,_ *_j_* represent the dimensional initial average surfactant concentration and dimensional surfactant concentration at maximum packing over the interface Γ_*j*_, respectively. Also, *R* is the ideal gas constant and *T* is the absolute temperature.

The governing equations (eqs 5–7) are discretized using the finite difference method on a uniform grid.^61^ The time step is selected based on the Courant-Friedrichs-Lewy (CFL) condition, as outlined by Sussman et al.^62^ Temporal integration is performed using the first-order forward Euler scheme. The no-slip and no-penetration boundary conditions are imposed on the left and right walls. The coupling between pressure and velocity is carried out through Chorin’s pressure projection method.^63^ The pressure Poisson equation is solved using the Successive Over-Relaxation method, with Neumann boundary conditions applied at the left and right walls. The pressure at the top wall is set to zero (*p_top_* = 0), and a steady/periodic pulsatile flow, as described in eq 1 is imposed at the bottom wall.

### Governing Equation for Surfactant Transport

In this study, we model the surfactant as insoluble, following the framework described by Leal.^60^ This implies that surfactant remains confined to the interface and does not exchange with the surrounding bulk fluid. This assumption is valid for systems where the adsorption/desorption kinetics are slow compared to convection at interface, or where the bulk concentration is sufficiently low. The resulting convection-diffusion equation along the interface allows us to focus on interfacial transport effects such as Marangoni stresses without the added complexity of bulk exchange kinetics. The dimensionless form of the insoluble surfactant convection-diffusion equation^64^ along the interface Γ_*j*_ can be written as

16 $$St\frac{\partial f_{j}}{\partial t}+u.\nabla f_{j}-n_{j}.\nabla u.n_{j}f_{j}=\frac{1}{Pe}\nabla_{s}^{2}f_{j}$$

The surface Laplacian operator $\nabla_{s}^{2}f_{j}$ is defined as^64^

17 $$\nabla_{s}^{2}f_{j}=\nabla^{2}f_{j}-\frac{\partial^{2}f_{j}}{\partial {n_{j}}^{2}}-\kappa_{j}\frac{\partial f_{j}}{\partial n_{j}}$$

Here, $Pe=\frac{g^{1/2}{\mathrm{D̃}}_{s}^{3/2}}{{\mathrm{D̃}}_{f}}$ is the Péclet number, D̃*_f_* is the surfactant diffusivity and we assume a constant surface diffusivity for the insoluble surfactant on both interfaces. Time discretization of the convection-diffusion equation (eq 16) is carried out using the two-step semi-implicit modified Crank–Nicolson scheme^34,64^ as described in eq 19, which offers second-order temporal accuracy and is stable under the condition Δ*t* = *O*(Δ*x*). The backward Euler method is applied for the first time-step alone,^34,64^ and is given by

18 $$St\left(\frac{f_{j}^{1}-f_{j}^{0}}{dt}\right)=\frac{\nabla^{2}f_{j}^{1}}{Pe}-{[-\frac{1}{Pe}\left(\kappa_{j}\frac{\partial f_{j}}{\partial n_{j}}+\frac{\partial^{2}f_{j}}{\partial n_{j}^{2}}\right)-u.\nabla f_{j}+n_{j}.\nabla u.n_{j}f_{j}]}^{0}$$

From the second time step onward, we use eq 19

19 $$\begin{array}{ll} St\left(\frac{f_{j}^{m+1}-f_{j}^{m}}{dt}\right) & =\frac{\nabla^{2}f_{j}^{m+1}-\nabla^{2}{f_{j}}^{m}}{2Pe}\\ & +\frac{3}{2}{[-\frac{1}{Pe}\left(\kappa_{j}\frac{\partial f_{j}}{\partial n_{j}}+\frac{\partial^{2}f_{j}}{\partial n_{j}^{2}}\right)-u.\nabla f_{j}+n_{j}.\nabla u.n_{j}f_{j}]}^{m}\\ & -\frac{1}{2}{[-\frac{1}{Pe}\left(\kappa_{j}\frac{\partial f_{j}}{\partial n_{j}}+\frac{\partial^{2}f_{j}}{\partial n_{j}^{2}}\right)-u.\nabla f_{j}+n_{j}.\nabla u.n_{j}f_{j}]}^{m-1} \end{array}$$

### Local Level-Set Method for Interface Capture and Surfactant Transport

In this study, we adopt the Level-Set Method to track the interfaces of the surfactant-laden compound droplet. Compared to Volume of Fluid (VOF) or Front Tracking methods, LSM offers a smooth, implicit interface representation with accurate curvature and unit normal vector computations essential for modeling interfacial tension and Marangoni effects. Additionally, its natural handling of topological changes and compatibility with Eulerian flow solvers makes it well-suited for analyzing complex droplet dynamics.^65–67^ In the traditional approach, the PDE governing the level set function is solved over the entire domain, which can be computationally expensive, especially in large domains where most of the region does not interact with the interface. The local level set method addresses this inefficiency by restricting calculations to a narrow band around the interface (a small neighborhood where the level set function has values close to zero). By doing so, it maintains accuracy near the interface while reducing unnecessary calculations far from it, making the method more efficient.^64,68–70^

In our numerical algorithm, we construct three tubes around the interface with widths γ_1_ < γ_2_ < γ_3_ corresponding to progressively larger regions of influence around the interface. The mathematical description of these tubes is given as

20 $$\begin{array}{l} T_{j_{1}}=\left\{\left(x_{p},y_{q}\right):\left|\phi_{j}\left(x_{p},y_{q}\right)\right|<\gamma_{1}\right\}\\ T_{j_{2}}=\left\{\left(x_{p},y_{q}\right):\left|\phi_{j}\left(x_{p},y_{q}\right)\right|<\gamma_{2}\right\}\\ T_{j_{3}}=\left\{\left(x_{p},y_{q}\right):\underset{l,k=-1,0,1}{\min}\left|\phi_{j}\left(x_{p+l},y_{q+k}\right)\right|<\gamma_{3}\right\} \end{array}$$

The widths, γ_1_, γ_2_, γ_3_ for constructing tubes around the interfaces (Γ*_os_* and Γ*_sc_*) are typically chosen as multiples of the grid size *dx*, to ensure alignment with the spatial discretization. Their values depend on the stencil size of the algorithm, with the widest stencil determining the outermost width of tube. For instance, in a fifth-order WENO scheme,^71^ which uses a 5-point stencil, γ_3_ must be wide enough to cover at least but not limited to two grid points on either side of the interface ensuring accurate resolution of the interface while encompassing smooth transitions and capturing the effects of the numerical stencil. This design balances accuracy, stability, and computational efficiency.

We choose γ_1_ = 6*dx*, γ_2_ = γ_1_ + 3*dx*, γ_3_ = γ_2_ + 3*dx*. These widths ensure that the tubes progressively expand outward from the interface while maintaining a structured relationship. We solve eq 16 as discussed earlier to get $f_{j}^{m+1}$ in tube $T_{j_{1}}$ , where *m* + 1 represents the next time step. The spatial discretization in eqs 18 and 19 involves not only points in tube $T_{j_{1}}$ but also points outside of $T_{j_{1}}$ , near the boundary of $T_{j_{1}}$ . To obtain the values of *f_j_* in a larger tube $T_{j_{2}}$ , we impose the Dirichlet boundary condition for $f_{j}^{m+1}$ at those points using extrapolation^64^ as

21 $$f_{j}^{m+1}=2f_{j}^{m}-f_{j}^{m-1},,\forall \left(x_{p},y_{q}\right)\in T_{j_{2}}-T_{j_{1}}$$

The evolution of the interfaces Γ*_os_* and Γ*_sc_* is modeled by advecting the corresponding level-set functions, *ϕ_os_* and *ϕ_sc_*, respectively, using the velocity fields obtained by solving eqs 5–7. This is achieved by solving the level set advection equation as described below.^64,68^

22 $$St\frac{\partial \phi_{j}}{\partial t}+C_{m}\left(\phi_{j}\right)v.\nabla \phi_{j}=0$$

with *j* = *os and sc* fluid pairs, where

23 $$c_{m}\left(\phi_{j}\right)=\{\begin{array}{ll} 0 & if\;\left|\phi_{j}^{m}\right|>\gamma_{2,}\\ \frac{{\left(|\phi_{j}^{m}|‐\gamma_{2}\right)}^{2}\left(2\left|\phi_{j}^{m}\right|+\gamma_{2}‐3\gamma_{1}\right)}{{\left(\gamma_{2}‐\gamma_{1}\right)}^{3}} & if\;\gamma_{1}<\left|\phi_{j}^{m}\right|\leq \gamma_{2}\\ 1 & if\;\left|\phi_{j}^{m}\right|\leq \gamma_{1.} \end{array}$$

Here, *c_m_* (*ϕ_j_*) is the cutoff function used to avoid discontinuities in the coefficients and numerical oscillations at the boundary^64,68^ of tube $T_{j_{2}}$ . Equation 22 is solved in tube $T_{j_{2}}$ .

Further, the level-set function ϕ*_j_* is reinitialized to regain its property as a distance function. Thus, eq 24 is solved in tube $T_{j_{3}}$ .

24 $$\frac{\partial \phi_{j}}{\partial \tau }-S\left(\phi_{j,0}\right)\left(1-\left|\nabla \phi_{j}\right|\right)=0$$

where

$$S\left(\phi_{j,0}\right)=\frac{\phi_{j,0}}{\sqrt{\phi_{j,0}^{2}+dx^{2}}}$$

In eq 24, *ϕ*_*j,*0_ denotes the ϕ*_j_* at τ = 0 and *S*(*ϕ_j_*,_0_) represents the sign function of *ϕ_j,0_*. Equations 22 and 24 are solved using a fifth-order WENO spatial discretization coupled with a second-order Runge–Kutta temporal integration.^47,59^ The zero Neumann condition is employed at the channel inlet, outlet, and the walls.

Next, the surfactant concentration *f_j_* is extended off the interface to ensure it is orthogonal to the level set function *ϕ_j_* within the vicinity of the interface.^68,70,72^ This is modeled by solving a simple linear convection equation (eq 25) in tube $T_{j_{2}}$ until steady state.

25 $$\{\begin{array}{l} f_{j\tau }+S\left(\phi_{j,0}\right)n_{j}.\nabla f_{j}=0,\\ f\left(x,0\right)=f_{0}\left(x\right) \end{array}$$

The extension process ensures that *f*(***x***,τ) = *f*_0_(***x***) if *ϕ*(***x***) = 0 along the interface. A second-order central difference scheme is used to compute ***n**_j_* while a fifth-order WENO scheme is employed to compute ∇*f* . Since the solutions at two successive time steps are nearly identical, only a limited number of iterations are required for the extension. The surfactant mass conservation step is employed as mentioned in literature.^34^

As the interface moves, the grid points in each tube need to be dynamically updated to reflect the new position. This involves adding grid points that newly satisfy the conditions for inclusion in a tube and removing points that no longer meet the criteria. The updates rely on recalculating |*ϕ_j_* | at each grid point and re-evaluating whether it falls within the bounds defined by γ_1_, γ_2_ and γ_3_. The reconstruction of the three tubes around the new interface from the previous ones is done by

26 $$\begin{array}{l} T_{j_{1}}\leftarrow \left\{\left(x_{p},y_{q}\right):\left|\phi_{j}^{m+1}\right|<\gamma_{1},\left(x_{p},y_{q}\right)\in T_{j_{3}}\right\}\\ T_{j_{2}}\leftarrow \left\{\left(x_{p},y_{q}\right):\left|\phi_{j}^{m+1}\right|<\gamma_{2},\left(x_{q},y_{q}\right)\in T_{j_{3}}\right\}\\ T_{j_{3}}\leftarrow \left\{\left(x_{p},y_{q}\right):\left|\phi_{j}^{m+1}\right|<\gamma_{3},\left(x_{p},y_{q}\right)\in T_{j_{3}}\right\}\cup \left\{\left(x_{p+l},y_{q+k}\right),l,k=-1,0,1:\left|\varphi_{j}^{m+1}\right|{<\gamma }_{3},\left(x_{p},y_{q}\right)\in T_{j_{3}}\right\} \end{array}$$

To summarize the numerical algorithm, the fluid flow eqs (eqs 5–7) are solved to compute the velocity field at the current time step (*t* = *m*). Thereafter, the updated velocity field is employed to solve the level-set advection equation (eq 22), in tube $T_{j_{2}}$ , which governs the motion of the interface. Next, the level-set functions are reinitialized by solving eq 24 in tube $T_{j_{3}}$ to maintain the signed distance property of the level-set function. The updated level-set function is used to determine unit normal vectors and curvatures to be used in the surfactant transport equation. Using the computed velocity field, the surfactant transport equation (eq 16) is solved in tube $T_{j_{1}}$ . The surfactant concentration is extended off the interface by solving eq 25 in tube $T_{j_{2}}$ . Based on the reinitialized level-set function *ϕ_j_*, the Heaviside function, physical properties (density, viscosity) and the tube geometries are recomputed to be used for fluid flow calculations in the next time step, (*m* + 1).

The cross-sectional average inlet velocity of the outer fluid (*V_in_*), as well as the average absolute velocities in the shell (*V_s_*) and core (*V_c_*) are determined at every time instant using the trapezoidal rule. All simulations maintain mass conservation with losses below 1%. The numerical implementation is developed in MATLAB 2024b and executed on a Dell Precision 3660 workstation with a Core i9 processor and 128 GB RAM.

## Model Validation

In the previous work,^48^ we have demonstrated the accuracy and reliability of the in-house level set-based solver through extensive validation tests. This includes comparing the steady state shape of a compound droplet with experiments reported by Zhang et al.^53^ Building on that foundation, the current work incorporates the effects of surfactants. To validate the extended model, we present three benchmark studies: (i) surfactant transport in a stationary droplet, which assesses the solver’s ability to handle pure diffusion-driven mass transport; (ii) surfactant transport in a translating droplet, validating the full numerical implementation of the surfactant transport equation; and (iii) the deformation of a surfactant-laden droplet subjected to shear flow, showcasing the solver’s ability to accurately resolve interfacial dynamics influenced by surfactants through the coupled implementation of mass conservation, momentum transport, and surfactant transport equations using the local level-set method. The first two validation tests along with the time step convergence tests are presented in the Supporting Information.

### Dynamics of Surfactant Laden Droplet under Shear Flow

This validation test involves a circular droplet of unit diameter subjected to a linear shear flow, with the far field boundary condition defined as ***u*** = *ye_x_*, where *e_x_* is the coordinate vector in *x*-direction, following the setup of Xu et al. 2012.^34^ The simulation domain spans [−5, 5], [−2, 2] and is periodic in *x*-direction. Under the influence of shear, the droplet experiences significant deformation. To simulate this behavior, the in-house solver couples the mass conservation, momentum balance, and surfactant transport equations within the local level set framework, as detailed in the Model Development. Gravity term is neglected. The droplet shapes at time *t* = 9 are shown in Figure 2 for three different surfactant coverages: ξ = 0 (clean droplet), ξ = 0.3 and 0.6. The blue solid curves represent results from our in-house solver, while the red dashed curves correspond to the reference data from Xu et al. 2012.^34^ The simulation parameters used in this study are *Ca* = 0.1, *β* = 0.2, *Pe* = 10, *Re* = 10, *μ* = *ρ* = 1. The relevant dimensionless parameters are defined as per Xu et al., to be

$$Re=\frac{\rho_{1}a^{2}\mathrm{\gamma ̣}}{\mu_{1}},Ca=\frac{\mu_{1}a\mathrm{\gamma ̣}}{\sigma_{o}},Pe=\frac{a^{2}\mathrm{\gamma ̣}}{D_{s}},\beta =\frac{RTf_{\infty }}{\sigma_{o}},\xi =\frac{f_{e}}{f_{\infty }},\rho =\frac{\rho_{2}}{\rho_{1}},\mu =\frac{\mu_{2}}{\mu_{1}}$$

where, *a* is radius of the droplet, γ ˙is the shear rate, *ρ* and *μ* are the ratio of density and viscosity of droplet phase to continuous phase. As the surfactant coverage ξ increases, the interfacial tension at the droplet tips decreases due to surfactant accumulation, leading to greater droplet deformation. At the same time, higher ξ intensifies Marangoni stresses, which act to resist this deformation by driving surfactant redistribution along the interface. Despite this opposing effect, the overall deformation of the droplet increases with increasing ξ. Figure 2 demonstrates that our in-house solver accurately reproduces the droplet shape and surfactant concentration profiles across varying surfactant coverages, thereby confirming the robustness and reliability of the numerical implementation.

**Figure 2:**
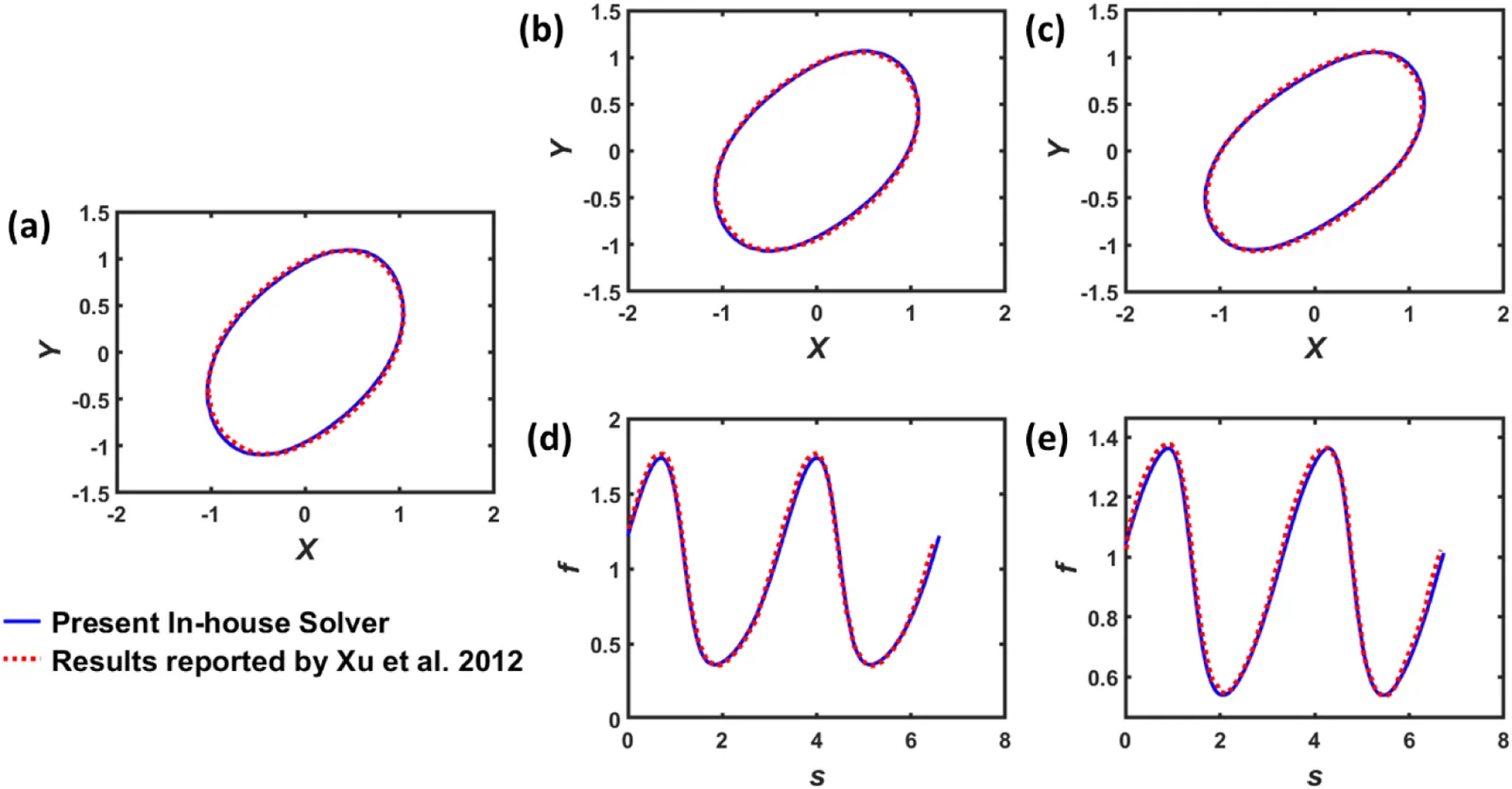
Dynamics of a surfactant-laden droplet under an applied shear flow. Comparison of our in-house solver (solid blue curve) with the benchmark results of Xu et al. 2012 ^34^ (red dot curve). Droplet shapes at *t* = 9 for different surfactant coverage ξ, i.e., (a) ξ = 0 (b) ξ = 0.3 (c) ξ = 0.6. Surfactant concentration as a function of arc length, s, for (d) ξ = 0.3 (e) ξ = 0.6. Grid size: 251 × 101. The arc length begins near the lower-left tip of the droplet.

### Grid Independence Test

The grid independence test is performed to assess how grid resolution affects the numerical solution and to determine the appropriate grid size required for accurate results. In this study, a compound droplet carrying an insoluble surfactant on both its shell and core interfaces is examined within a vertical channel extending over [−0.5405, 0.5405] in the *x*-direction and [0, 5.4054] in the *y*-direction. The droplet is initially positioned at [0, 0.6] in all the numerical simulations, unless stated otherwise. While the grid independence test was performed for both steady and pulsatile inflows over a range of parameters, the results reported here correspond to pulsatile inflows, for three different grid sizes: 41 × 201, 61 × 301, and 81 × 401. The droplet shape and shell velocity (*V_s_*) vs time are depicted in Figure 3a and Figure 3b, respectively, while the corresponding surfactant concentration on the outer-shell (*f_os_*) and shell–core (*f_sc_*) interfaces as a function of arc length, *s* are plotted in Figure 3c and Figure 3(d), respectively. Under pulsatile inflow, the velocity increases from zero and oscillates about the mean, exhibiting both positive and negative values that indicate back-and-forth droplet motion due to the pulsatile forcing. While the droplet position shows a marginally smaller translation and a higher *f_sc_* on the coarsest grid (41 × 201), the droplet position, shape, the average shell velocity and surfactant concentrations show good agreement for the grid sizes, 61 × 301 and 81 × 401. Therefore, a grid size of 81 × 401 is adopted for all subsequent simulations unless otherwise specified.

**Figure 3:**
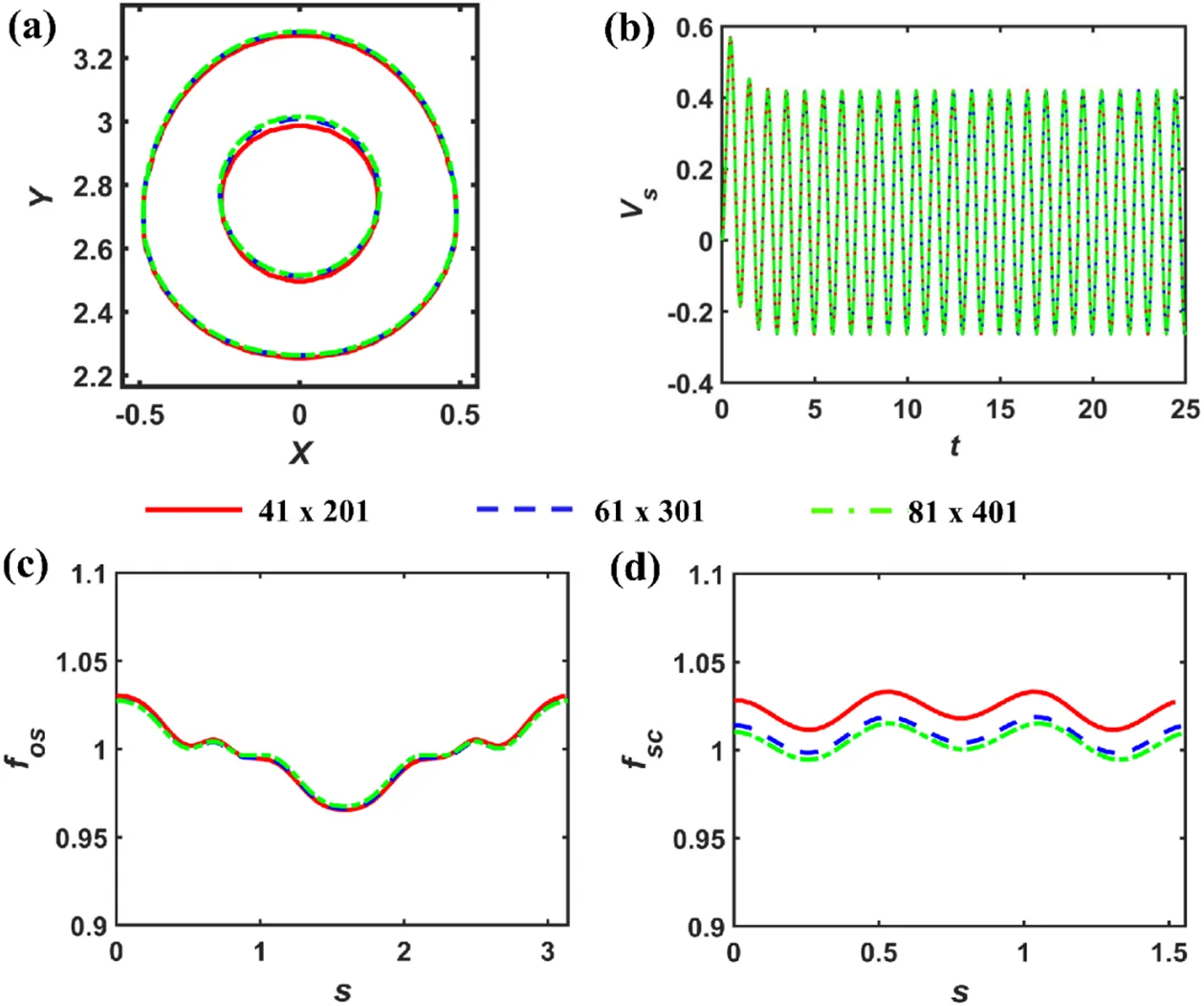
Grid independence test. Compound droplet position under pulsatile flows at dimensionless time*t* = 25. Plot of (a) droplet position, b) average velocity of shell (*V_s_*) vs time, surfactant concentration vs arc length for the (c) outer-shell and (d) shell–core interfaces. The simulation parameters are Rc = 0.5 *R_s_*, *Bo_os_* = *Bo_sc_* = 1,*Re = 10,* μ̂*_s_* = μ*^_c_* = 1, ρ̂*_s_* = 0.9, ρ̂*_c_* = 1, Δ*p* = 0.5, ξ = 0.6, *β* = 0.5, *Pe* *= 10*, *St* = 1 and *A* = 1.

## Results and Discussion

### Dynamics of a Surfactant-Laden Compound Droplet under Steady Poiseuille Flow

The dynamics of an insoluble surfactant-laden, confined compound droplet under an imposed steady flow is first investigated. As outlined in the model development, three scenarios of initial surfactant distribution are considered: (i) surfactant present only on the shell interface (C1), (ii) surfactant confined only to the core interface (C2), and (iii) surfactant present on both shell and core interfaces (C3). The results are compared with those of a clean compound droplet (C) as depicted in Figure 4. Initially, the surfactant concentration is uniform for all the three scenarios of surfactant distribution.

**Figure 4:**
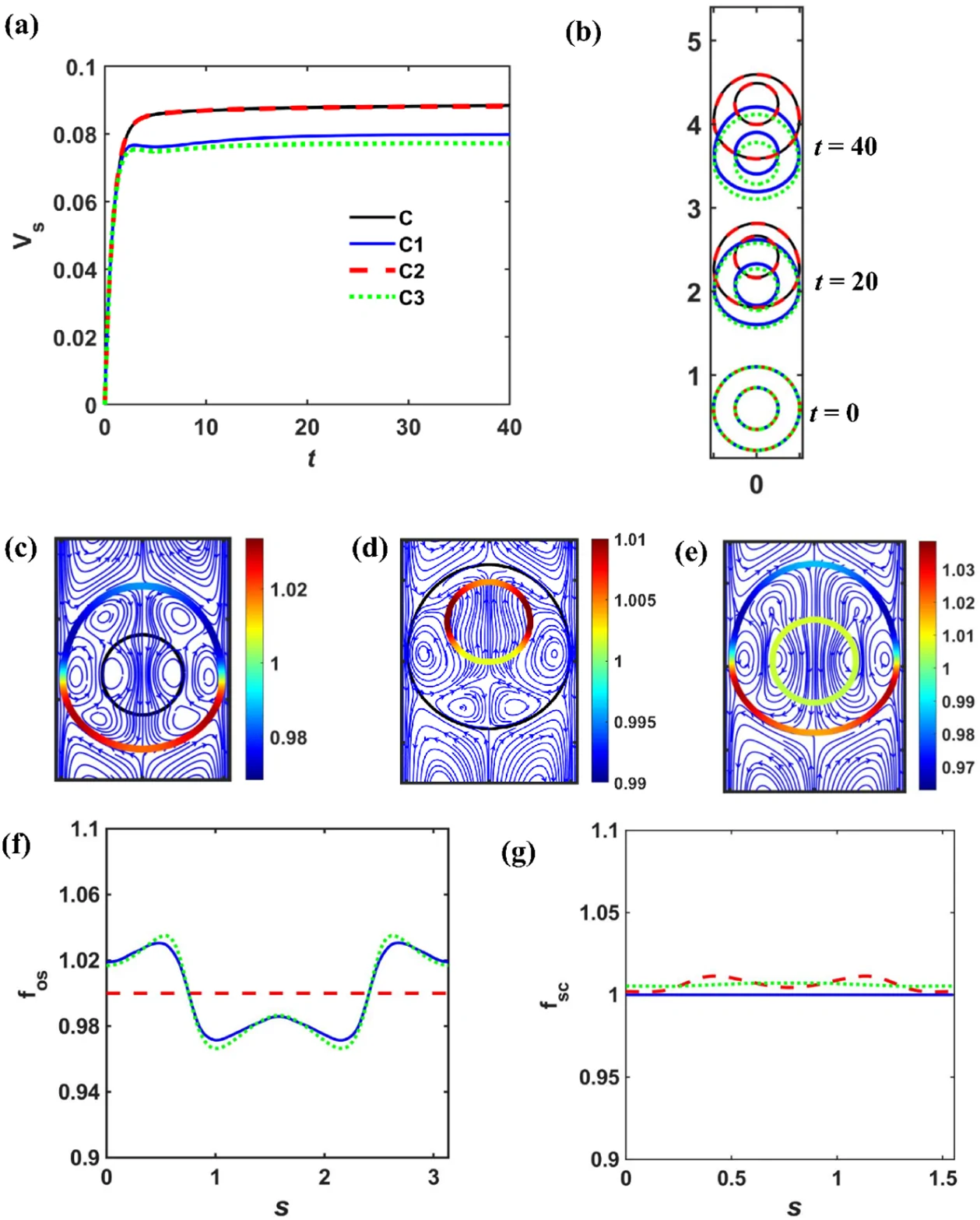
(a) Temporal evolution of the shell velocity (*V_s_*) of a compound droplet for four cases: clean (C), surfactant present on the shell interface (C1), surfactant present on the core interface (C2), and surfactant present on both shell and core interfaces (C3). (b) Temporal snapshots showing droplet position, deformation and interface evolution at *t* = 0, 20, and 40. Panels (c, d, e): Streamlines and surfactant concentration distributions at steady state (*t* = 40), showing interfacial flow circulation patterns within the core and shell for (C1), (C2), and (C3). Panels (f, g): Interfacial surfactant concentration distribution (*f_os_* and *f_sc_* along the arc length *s* for cases (C1), (C2) and (C3), respectively. The other simulation parameters are *Ar* = 10, *Pe* = 10, Δ*p* = 0.5, ξ = 0.6, *β* = 0.5, *Bo_os_* = *Bo_sc_* = 1, ρ̂*_o_* = 1, ρ̂*_^s_* = 0.9, ρ̂*_c_* = 1, μ̂*_o_* = 1, μ̂*_s_* = 1, μ̂*_c_* = 1, *R_s_* = 0.925 *R* and *R_c_* = 0.5 *R_s_* where *R, R_s_, R_c_* represent the dimensionless half-width of channel, radius of the shell and core, respectively.

Figure 4a illustrates the plot of shell velocity (*V_s_*) vs time for all four cases. As observed for the case of a simple droplet, the clean compound droplet translates faster than a surfactant-laden compound droplet. When the droplet is clean (case (C)), the shell velocity reaches a steady value of 0.0885 as observed in Figure 4a. For case (C1), a significant drop in the shell velocity is observed (*V_s_* = 0.0798), consistent with the behavior reported for a single droplet.^19^ In contrast, case (C2) results in a velocity identical to that of the clean case (*V_s_ =* 0.0883). This behavior arises because the core is significantly smaller and remains confined within the outer shell and is relatively isolated from the externally imposed flow. For case (C3), the droplet is significantly retarded, and the translational velocity is the lowest (*V_s_ =* 0.0772). Furthermore, Figure 4b illustrates the droplet shape and position at various times for all scenarios of surfactant distribution. At early times, the difference between the clean and surfactant-laden cases are small, while at later times, it is evident that the translation speed of the compound droplet follows the order (C) ∼ (C2) >;(C1) > (C3). The percentage relative difference in the shell velocity for cases (C1), (C2) and (C3) with respect to the clean droplet (C) are 9.8%, 0.19% and 12.7%, respectively. This indicates that the presence of surfactant on the shell has a pronounced effect on its translational speed whereas, the presence of surfactant on the core has little influence as the core fluid remains encapsulated, thereby having minimal interaction with the surrounding flow.

Figures 4c–4e provide additional insights into the hydrodynamics and interfacial behavior by depicting streamlines and interfacial concentration distributions at steady state. The blue streamlines represent the internal circulation patterns, while the color map denotes the local surfactant concentration along the interfaces. These figures correspond to the three cases of initial surfactant distributions, (C1), (C2) and (C3), respectively. The color contours denote normalized surfactant concentration, where deviations from unity correspond to local accumulation (red) or depletion (blue) zones. Further, Figure 5a–5c compare the distribution of interfacial tension, Marangoni forces and interfacial velocity respectively, for cases (C1) and (C2). For ease of comparison, all interfacial quantities are plotted as functions of the normalized arc length *s*′, where *s*′ is the ratio of arc length (*s*) to the total length of the interface. Accordingly, *s*′= 0(0.5) at the rear (front) pole.

**Figure 5:**
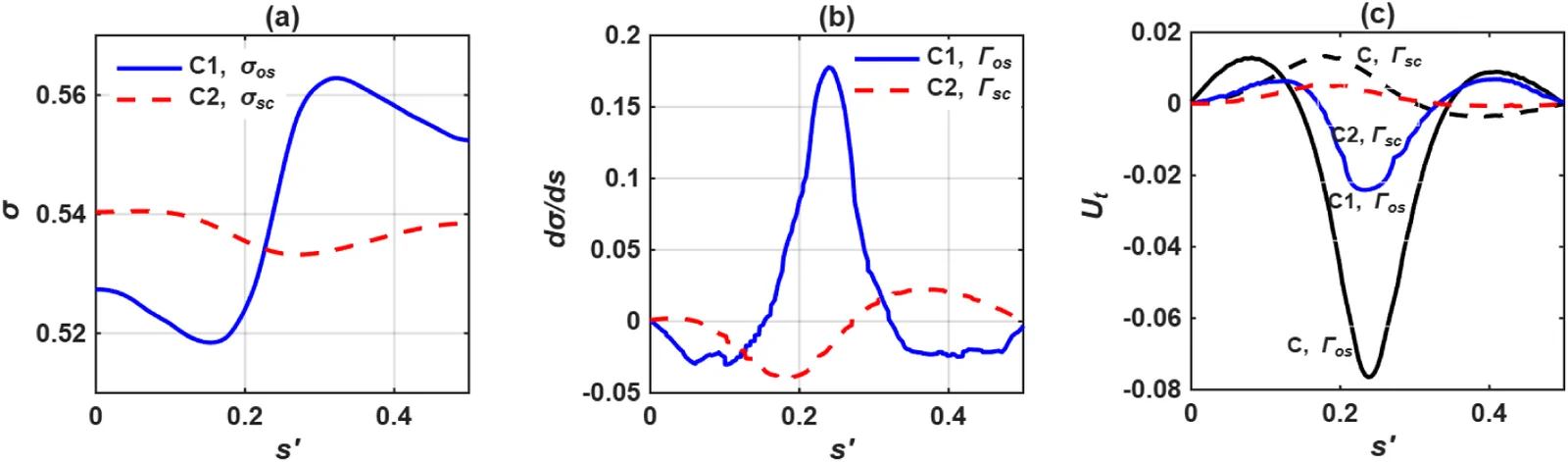
(a) Variation of interfacial tension as a function of the normalized arc length for cases (C1) and (C2). (b) Corresponding Marangoni stress distributions along the interface, plotted from the rear to the front pole of the droplet. (c) Tangential interfacial velocity, measured in the droplet reference frame, as a function of the normalized arc length for cases (C1) and (C2), together with the corresponding profile for the clean droplet (C). The simulation parameters are identical to those used in Figure 4.

We begin our discussion with case (C1), where surfactant is initially distributed over the shell interface. Unlike a simple droplet, a clean compound droplet consists of multiple fluid–fluid interfaces and consequently exhibits a more complex internal circulation pattern under an imposed external flow. The flow field consists of a pair of primary vortices and two pairs of secondary vortices within the shell fluid, together with a pair of recirculating vortices inside the core.^40–42^ The addition of surfactant on the shell interface (case (C1)) does not alter this overall vortex topology, and the streamline pattern shown in Figure 4c remains qualitatively similar to that of the clean compound droplet. In case (C1), the upward flow of the droplet leads to the advection of the insoluble surfactant toward the south pole (rear of the shell), leading to maximum surfactant accumulation (*f_os_∼* 1.02) close to the rear and minimum concentration (*f_os_* ∼ 0.98) close to the front. This behavior is depicted in the color map of Figure 4c and the surfactant concentration vs arc length plot in Figure 4f.

According to the Langmuir equation of state, the increase in surfactant concentration reduces the local interfacial tension. Consequently, the interfacial tension reaches its minimum near the rear of the shell (*s*′∼ 0.16) and its maximum near the front (*s*′∼ 0.32), as shown in Figure 5a. The resulting interfacial tension gradient generates Marangoni stresses, which act tangentially along the interface from regions of low interfacial tension (high surfactant concentration) toward regions of high interfacial tension (low surfactant concentration). Figure 5b reveals the corresponding variation of Marangoni stress as a function of normalized arc length *s*′. The Marangoni stress is zero at the rear pole and remains negative up to *s*′∼ 0.16 reflecting a monotonic reduction in the interfacial tension as depicted in Figure 5a. Consequently, the Marangoni flows is directed from *s*′∼ 0.16 to *s*′ = 0, in the backward direction. Between *s*′∼ 0.16 to 0.32, the Marangoni stress remains positive reaching a maximum of about 0.18 at around *s*′∼ 0.23, and drives the flow from rear toward front. Beyond *s*′∼0.32, the Marangoni stress becomes negative and approaches zero at the front pole, resulting in another region of backward-directed interfacial flow.

To further elucidate the influence of Marangoni stresses on the interfacial dynamics, Figure 5c compares the tangential interfacial velocity, *U_t_*, measured in a reference frame moving with the droplet, for cases (C) and (C1). The presence of surfactant significantly suppresses the magnitude of the tangential interfacial velocity compared with the clean droplet, although the overall velocity distribution remains qualitatively similar. The tangential velocity is zero at both the rear and front stagnation points. For case (C1), from the rear pole to approximately *s*′∼ 0.16, the interface exhibits an upward tangential motion, consistent with the streamline pattern shown in Figure 4c. Beyond this location, the tangential velocity changes sign, indicating flow reversal associated with the primary recirculating vortex within the shell. Around *s*′∼ 0.32, the tangential velocity again becomes positive, corresponding to the secondary vortex located near the front of the droplet. These recirculating flow structures along with the external fluid flow transport surfactant from the front toward the rear of the shell interface, producing the observed maximum and minimum surfactant concentrations at *s*′∼ 0.16 and *s*′∼ 0.32, respectively. Therefore, the Marangoni-induced interfacial flow is directed opposite to the surfactant convection caused by the imposed external flow, thereby significantly retarding the droplet (Figure 4a and **4b**). This behavior aligns with previously reported observations for single droplets containing insoluble surfactants.^19^ A similar observation can be made for case (C3). Figure 4f shows the surfactant concentration (*f_os_*) vs arc length plot for cases (C1) and (C3) to nearly overlap, with maxima and minima occurring at similar locations. In contrast, when surfactant is initially present over the core interface as in cases (C2) and (C3), the color maps reveal that the concentration to peak at the lateral sides (east and west) of the core and is minimal near the rear. Figure 4g reveals the surfactant distribution on the core remains nearly uniform on the core interface. Hence, case (C2) presents only minor variations in interfacial tension (Figure 5a) and a significantly weaker Marangoni stress over the core interface (Figure 5b). Further, the tangential interfacial velocity on the core interface is much smaller in magnitude as compared to that on shell interface in case (C1), as depicted in Figure 5c. As a result, the presence of surfactant on the core produces only a marginal reduction in the droplet velocity compared with the clean case, as evident from Figure 4a and **4b**, confirming that surfactant confined to the inner interface has a relatively weak influence on the overall hydrodynamics of the compound droplet.

Since the surfactant concentration gradients are stronger on the outer (shell) interface—which is directly exposed to the external flow—than on the inner (core) interface, it can be concluded that the presence of surfactant on the shell has a much stronger influence on the overall droplet dynamics than its presence on the core. To examine whether these conclusions are influenced by the chosen compound droplet geometry, additional simulations comparing clean (C), surfactant on shell (C1) and surfactant on core (C2) were performed by varying the core to shell radius ratio over a physically relevant range, $R_{C-S}=\frac{R_{C}}{R_{S}}=$ 0.4, 0.5, 0.6, and 0.7. The results confirm that the above conclusions remain valid across the entire range of core sizes investigated. These additional results have been included in the Supporting Information.

Next, the droplet deformation is quantified for various cases of surfactant distribution and compared with that of a clean compound droplet. Figure 6a shows the temporal evolution of the deformation index (*DI*) for the compound droplet under different interfacial conditions. The *DI* defined as the ratio of the droplet length along the streamwise and transverse directions $\left(DI=\frac{L}{B}\right)$ , quantifies the droplet’s shape change under an imposed flow.^19^ Values of *DI* > 1 indicate elongation along the flow direction, whereas *DI* < 1 represents oblation or flattening of the droplet perpendicular to the flow. As shown in Figure 6a, *DI* is equal to unity at *t* = 0 and gradually reaches a steady value for all surfactant distribution scenarios. The curves in Figure 6a labeled C–S and C–C respectively denote the core and shell behavior in the clean configuration. These serve as the references for interfacial mobility and deformation. A similar notation is used for the other configurations as well. For the clean case, the droplet deforms under the imposed flow, and the deformation index of shell increases above unity, indicating elongation, while that of the core (labeled by C–C) drops below unity, indicating an oblate core as shown in Figure 6a. Next, when surfactant is present only on the shell (C1–S), it lowers the local interfacial tension at the outer interface rendering the shell more deformable. Hence the compliant shell shows a higher elongation (*DI* > 1.03) in comparison to its clean counterpart (C–S, where *DI* ∼ 1.015). Meanwhile, the core remains clean (C1–C), retaining a uniform interfacial tension and an almost circular shape (which arises due to the elongation of the surrounding shell) in contrast to its oblate deformation observed in case (C). Relative to the clean case (C), the shell and core deformation indices in case (C1) increase by 2.42% and 2.11%, respectively, indicating a slight increase in elongation.

**Figure 6:**
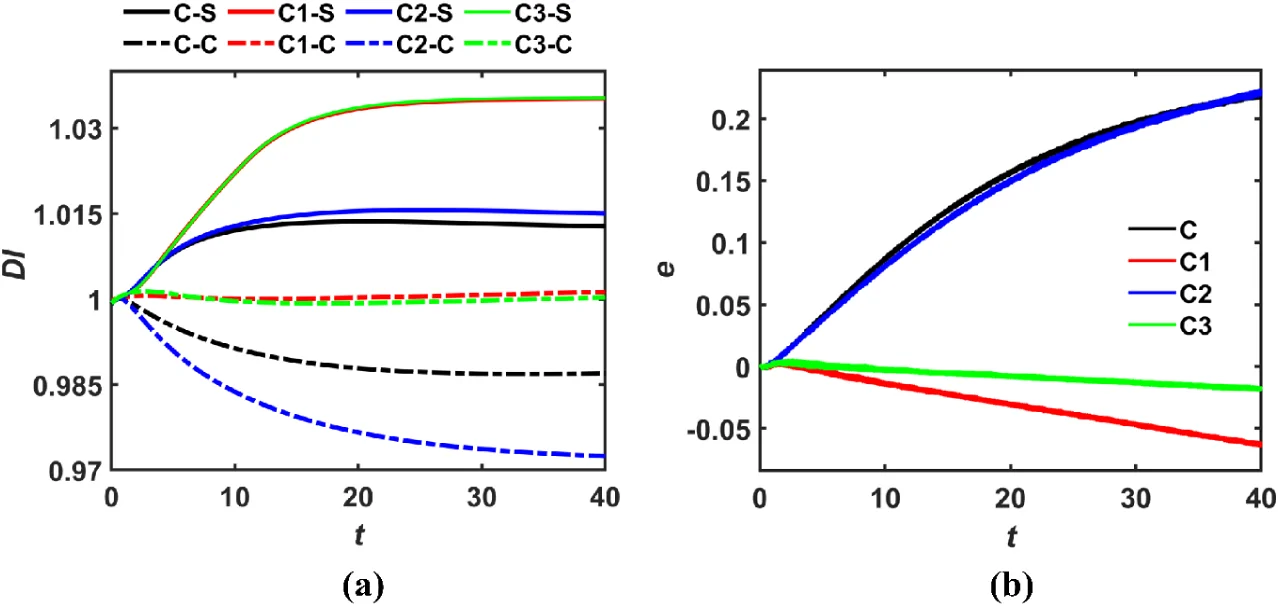
(a) Temporal variation of the deformation index (*DI*) for different cases: C–S (clean shell), C–C (clean core), and surfactant-laden interfaces (C1–S, C1–C, C2–S, C2–C, C3–S, C3–C), where C1, C2, and C3 denote surfactant on shell, core, and both interfaces, respectively. (b) Core eccentricity vs time plot for the four cases.

For case (C2), the presence of surfactant reduces the core’s effective interfacial tension, allowing it to deform more easily, as seen from C2–C. The shell experiences almost the same stress as in the clean case, so its deformation depicted by C2–S remains close to that of a clean shell (depicted by C–S). Thus, the presence of surfactant only on the core interface causes an enhanced oblation of the core in comparison to the clean case (C–S) (a 1.68% higher oblation in comparison to case (C)), while the shell elongation remains largely unaffected. Finally, for case (C3), the reduced interfacial tensions on both surfaces increase their compliance. As a result, the shell (depicted by C3–S) exhibits enhanced elongation (2.17% elongation compared to case (C)), while the core (depicted by C3–C) remains nearly circular, exhibiting deformation characteristics very close to the case when surfactant is only on the shell (C1–S).

Another interesting feature is the eccentricity of core, which is strongly influenced by the presence of surfactant. The eccentricity *e*, defined as the distance between the centroids of the shell and core, is plotted as a function of time in Figure 6b for different surfactant distribution scenarios. Initially, *e* is zero since the compound droplet is concentric. Under an imposed steady flow, the core of a clean compound droplet translates faster than the shell^42,48^ causing the eccentricity to increase with time. Hence, the core progressively reaches toward the front of the shell as indicated by Figure 4b. At *t* = 40, *e* ∼ 0.2159. A similar trend is observed when the shell is clean and the surfactant is present only on the core, case (C2) as shown in Figure 6b and Figure 4d.

In contrast, when the shell is laden with an insoluble surfactant, the compound droplet is significantly retarded due to Marangoni stresses. This reduction in the shell velocity also slows the motion of the core (which is heavier than shell in the present study) leading to a slight negative eccentricity (*e* ∼ −0.06), where the core shifts toward the rear of the shell as illustrated by Figure 6b and Figure 4c. A similar behavior is observed for case (C3), where the surfactant is distributed in both interfaces. In this case, the competing Marangoni stresses in the two interfaces result in a marginal negative eccentricity (Figure 6b and Figure 4e). While several previous studies have reported the occurrence of core pinch-off,^56^ the core velocity for the clean compound droplet considered in the present study is not high enough to cause pinch-off. Thus, compared to the clean case, the eccentricity decreases from 0.2159 to −0.0639 (C1) and −0.0190 (C3), indicating a reversal in the direction of core displacement, whereas case (C2) exhibits hardly any change. The presence of surfactants on the shell markedly reduces the core eccentricity, maintaining the core close to a centered position and potentially delaying the onset of core pinch-off.

### Dynamics of Surfactant-Laden Compound Droplet under a Pulsatile Inflow

This section explores the role of pulsatile inflow on the surfactant distribution and hence on the hydrodynamics of the compound droplet for various initial surfactant distribution scenarios. Figure 7 presents the dynamics of a compound droplet subjected to pulsatile flow, comparing clean and surfactant-laden conditions. Figure 7a compares the compound droplet position for all cases ((C), (C1), (C2), and (C3)) at times, *t* = 0, 20, and 40. As observed for a steady inflow, the presence of surfactant retards the droplet motion. As seen in Figure 7b, the imposed pulsation introduces a periodic acceleration and deceleration of the surrounding fluid. As a result, for the parameters chosen in this study, the shell exhibits alternating positive and negative velocities, leading the compound droplet to traverse back and forth for all surfactant distribution scenarios. The inset image in Figure 7b reveals that the shell velocities follow the order, (C) ∼ (C2) > (C1) > (C3), consistent with the trend observed under steady inflow. The main difference is that the velocities initially zero, gradually increase and oscillate about a mean value. This magnitude of shell velocities is directly reflected in the position of the compound droplet in Figure 7a.

**Figure 7:**
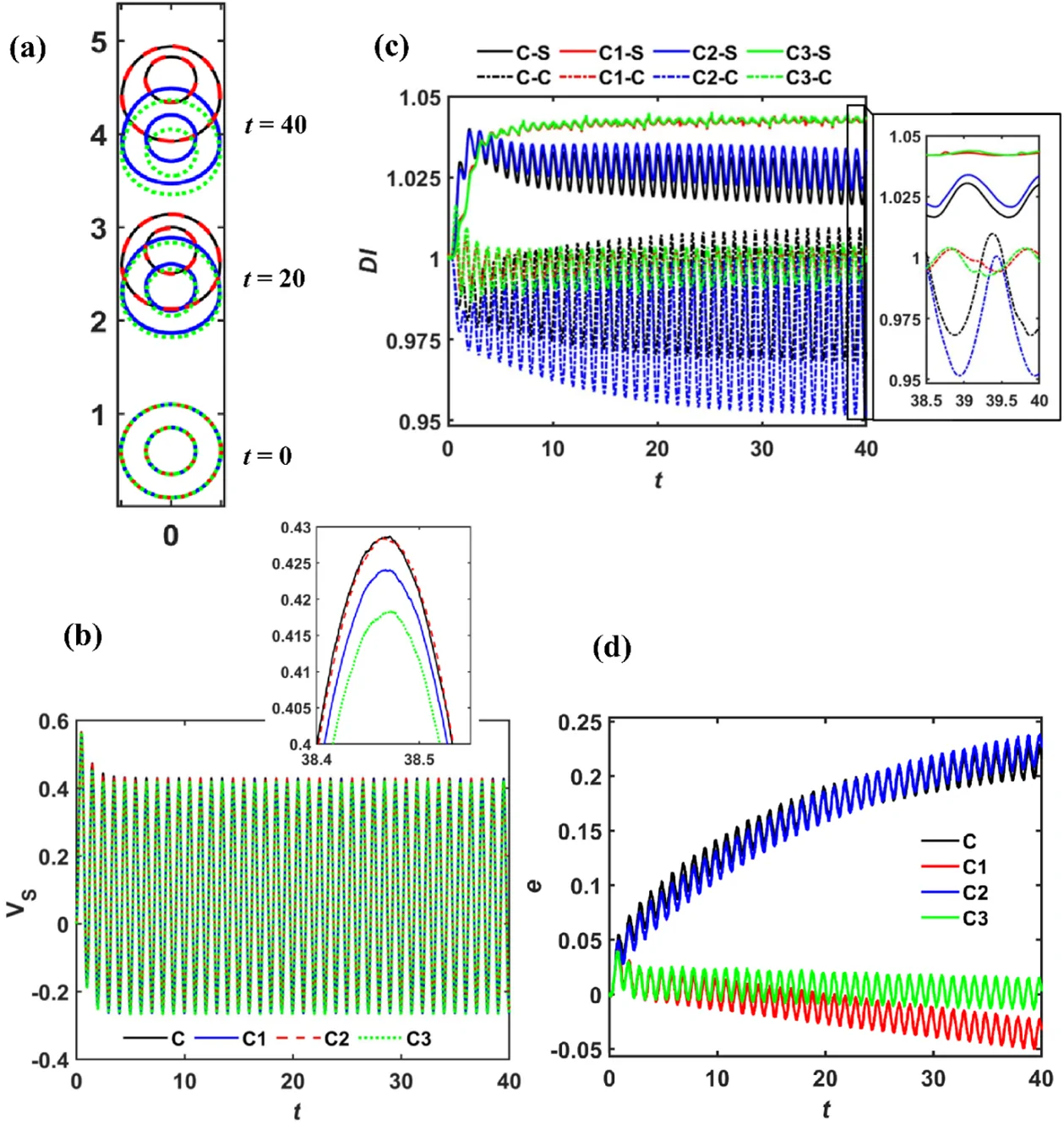
Dynamics of a compound droplet in pulsatile flow under both clean and surfactant-laden conditions. (a) Position of the compound droplet under various surfactant distribution scenarios at t = 0, 20, and 40. The solid black, solid blue, dashed-red and the dotted-green curves correspond to cases (C), (C1), (C2) and (C3) respectively. (b) Comparison of time-varying shell (Vs) velocities for the four cases (c) Comparison of time evolution of core and shell deformation indices for all cases. (d) Comparison of temporal evolution of core eccentricities for the four cases. The simulation parameters are *Ar* = 10, *Pe* = 10, Δ*p* = 0.5, *Bo_os_* = *Bo_sc_* =1, ρ̂*_o_* = 1, ρ̂ *_^s_* = 0.9, ρ̂*_c_* = 1, μ̂*_o_* = 1, μ̂*_s_* = 1, μ̂*_c_* = 1, *St* = 1, *A* = 2, *R_s_* = 0.925 *R* and *R_c_* = 0.5 *R_s_*.

Next, the time evolution of deformation indices of the core and shell interfaces are depicted in Figure 7c for various scenarios of initial surfactant distribution. Unlike in steady flows, where the values of *DI* reach a steady value, the imposed pulsatile forcing expectedly causes the compound droplet to develop time-dependent deformations, that oscillates about a mean value, although the overall trends remain broadly similar to those under a steady inflow. For a clean compound droplet, the shell assumes a prolate shape (indicated by *DI* > 1) while the core becomes oblate (*DI* < 1), with both interfaces continuously deforming about their respective mean values. For case (C1), the elongation of the compliant shell increases, which stretches the core along the axial direction resulting in a circular core. In contrast, for case (C2), the shell exhibits deformation similar to the clean case, while the core shows high-amplitude oscillations with magnitudes indicating enhanced oblation, suggesting that the reduced interfacial tension at the core interface increases the internal droplet deformation. Interestingly, the encapsulated core exhibits larger-amplitude deformations compared to the outer shell, even though the shell is directly exposed to the surrounding pulsatile forcing. In the pulsatile-flow case, the time-independent component of the imposed pressure drop generates a mean shear rate in the ambient fluid, which maintains a mean deformation of the shell interface. Consequently, the periodic component of the pressure drop must then compete with this mean shear-induced deformation, resulting in a relatively small-amplitude oscillatory deformation of the shell interface. In contrast, the core interface is relatively shielded from the effects of the mean shear and remains largely undeformed within the shell. It can therefore respond more readily to the periodic variations in the imposed pressure, resulting in a larger-amplitude oscillatory deformation. Similar to case (C1), case (C3) exhibits a greater shell elongation accompanied by reduced core oblation, as the elongating shell stretches the oblate core toward a nearly circular shape. Although the core undergoes larger amplitude time-dependent deformations in comparison to the shell under a pulsatile inflow, the overall hydrodynamics is primarily dictated by the presence of surfactant on the shell interface.

Figure 7d shows the temporal variation of core eccentricity under pulsatile inflow for the four cases, (C), (C1), (C2) and (C3). The eccentricity for cases (C) and (C2) nearly coincide and remain above zero, indicating that the core is positioned closer to the front of the shell, as also evident from Figure 8d and e. In contrast, for cases (C1) and (C3), the core remains approximately near the center, with a slight shift toward the rear in case (C3) and a more pronounced rearward bias in case (C1), similar to the observations made under a steady inflow. The corresponding compound droplet configurations for these two cases are depicted in Figure 8b–c, 8f–g.

**Figure 8:**
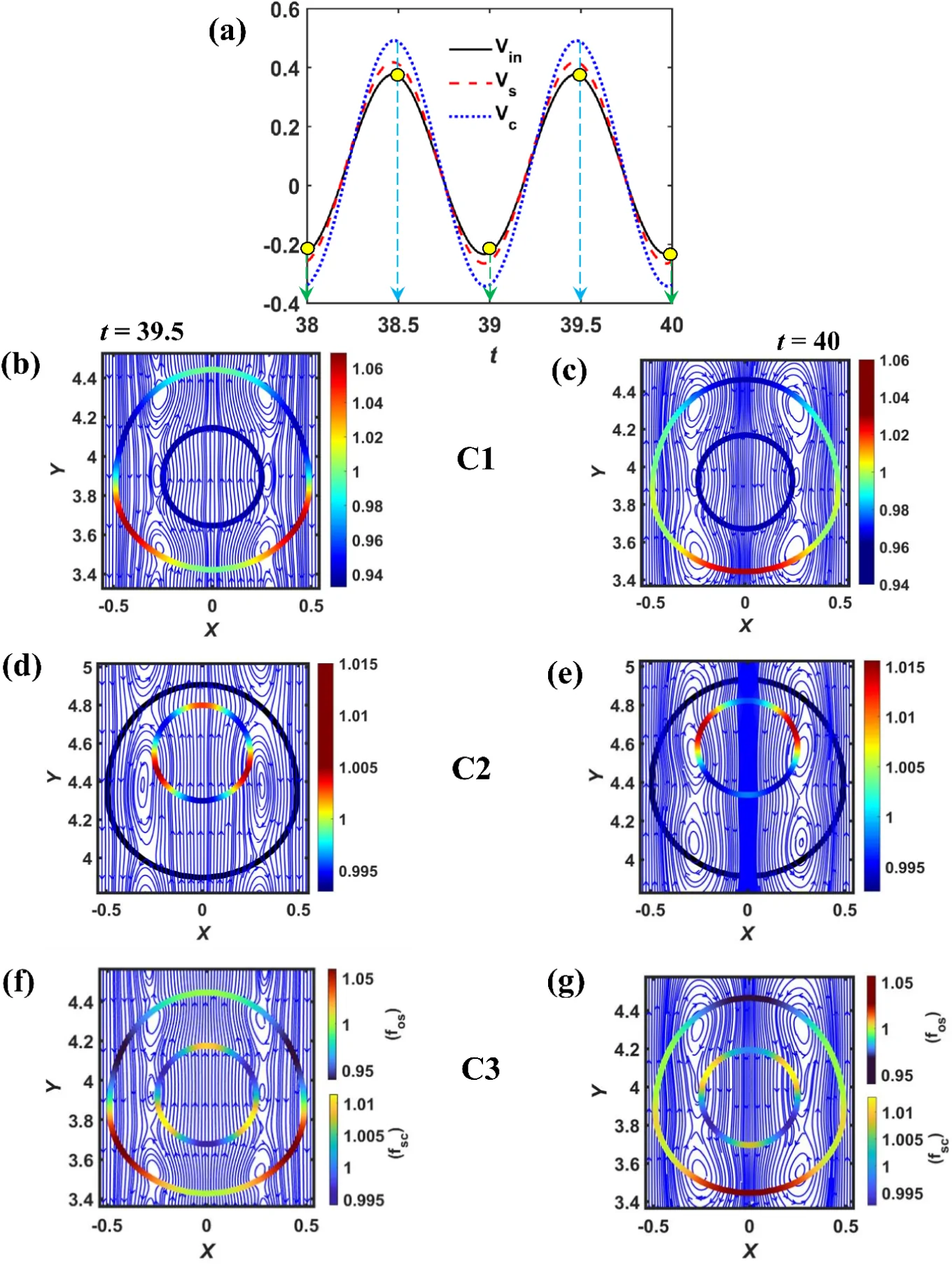
(a) Time-varying inlet (*V_in_*), shell (*V_s_*) and core (*V_c_*) velocities of a compound droplet in case (C3) showing a periodic flow response. (b–c), (d–e) and (f–g): Streamlines and color maps of interfacial surfactant concentration at *t* = 39.5, 40 for cases (C1), (C2) and (C3), respectively.

In steady flows, the shell always showed maximum surfactant accumulation near the rear corresponding to the stagnation region between the rear secondary vortex and the primary vortex, while the minimum concentration occurs near the front stagnation region between the front secondary vortex and the primary vortex. The resulting nonuniform surfactant distribution generates significant interfacial tension gradients and Marangoni stresses, thereby strongly influencing the droplet dynamics. However, adding surfactants to the core interface resulted in a nearly uniform surfactant distribution, resulting in weak Marangoni stresses and negligible influence on the hydrodynamics.

Under pulsatile inflow conditions, the periodic reversal of the inlet velocity gives rise to considerably more complex interfacial dynamics, which are analyzed through Figures 8 and 9. Figure 8 shows the contours of surfactant distribution and streamline patterns for cases (C1), (C2) and (C3) at various time instants. In Figure 8a, the cross-sectionally averaged inlet velocity (*V_in_*), mean shell velocity (*V_s_*), and mean core velocity (*V_c_*) for case (C3) exhibit synchronized oscillations for the parameters chosen in the study, reflecting the imposed pulsating flow. Consistent with our earlier works, the core—located farthest from the channel wall-exhibits the highest velocity followed by the shell and then the inlet velocity of the surrounding medium. The crests (*t* = 38.5, 39.5) and troughs (*t* = 38, 39, 40) over two oscillation cycles are identified and the color map of surfactant distribution at these instants are presented for the three cases, (C1), (C2) and (C3). Further, Figure 9 compares the surfactant concentration, interfacial tension, Marangoni stresses and the tangential interfacial velocities as a function of the normalized arc length *s′* for cases (C1) and (C2) at two time instants, *t* = 39.5 and 40, respectively.

**Figure 9:**
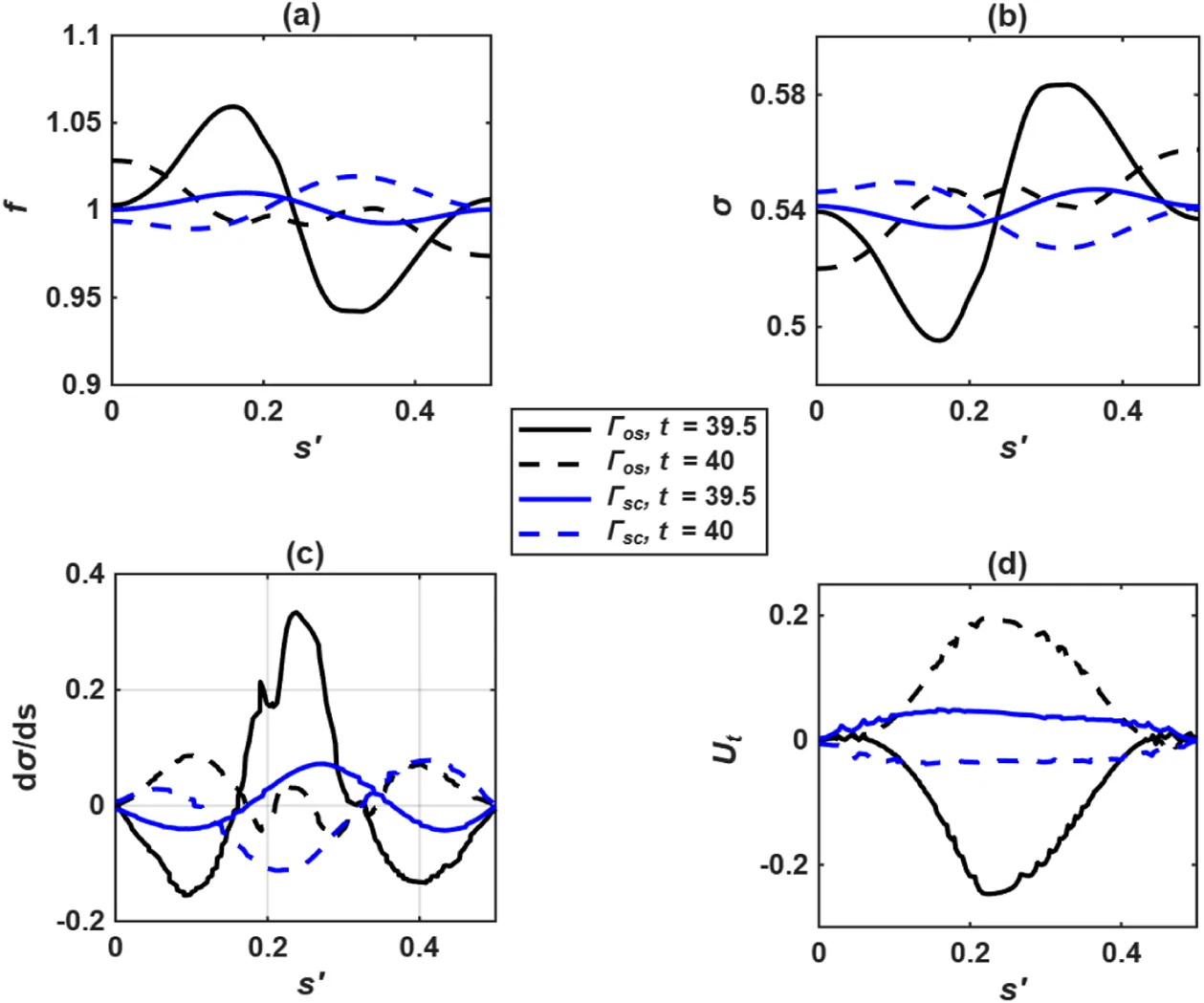
Comparison of the variation in (a) surfactant concentration, (b) interfacial tension, (c) Marangoni stress, and (d) tangential interfacial velocity along the outer-shell (Γ*_os_*) and shell–core (Γ*_sc_*) interfaces as functions of the normalized arc length, *s*′, for cases (C1) and (C2), respectively.

As seen for a steady inflow, Figure 8b and 8f corresponding to the velocity crest (*t* = 39.5) for cases (C1) and (C3) respectively, reveal a buildup of surfactant adjacent to the rear pole of the shell on its either side, and likewise there is minimum surfactant concentration close to the pole at the front. This behavior is further quantified in Figure 9a for case (C1), which depicts surfactant concentration vs normalized arc length, from rear to front pole of the droplet. As shown in Figure 9b, the corresponding interfacial tension attains a minimum near *s*′ ∼ 0.16 and a maximum around *s*′ ∼ 0.32, consistent with the steady-flow results. Consequently, the Marangoni stress distribution shown in Figure 9c is negative from the rear pole to *s*′ ∼ 0.16, becomes positive between *s*′ ∼ 0.16 and *s*′ ∼ 0.32, and again turns negative beyond this location. The resulting Marangoni flows are directed from regions of low interfacial tension (high surfactant concentration) toward regions of high interfacial tension (low surfactant concentration), thereby opposing the surfactant convection induced by the imposed flow and retarding the translational motion of the droplet. Unlike the steady-flow case, however, the streamline pattern in Figure 8b and the tangential interfacial velocity profile shown in Figure 9d is predominantly unidirectional, indicating a substantial suppression of interfacial recirculation during the velocity crest.

During the trough phase, when the shell velocity becomes negative (e.g., *t* = 40 in Figure 8a), the compound droplet undergoes reverse motion. This is reflected in the streamline pattern shown in Figure 8c, where the direction of the recirculating streamlines reverses in response to the flow reversal. While the outer fluid motion relative to the droplet is in the downward direction in the crest phase (Figure 8), it becomes upward in the trough (Figure 8c). As illustrated in Figure 9(a), the surfactant concentration remains maximum near the rear pole (*s*′ = 0) and minimum near the front pole; however, the variation in surfactant concentration along the interface is considerably weaker than that observed during the velocity crest. Consequently, the resulting interfacial tension gradients and Marangoni stresses are smaller, as shown in Figure 9c, although their direction reverses in accordance with the reversed flow. Accordingly, the tangential interfacial velocity in the droplet reference frame is predominantly directed upward along the interface, consistent with the reversed streamline pattern observed in Figure 8c. Thus, under pulsatile flow, the concentration gradients continually evolve as the shell continuously deforms, leading to periodic redistribution of surfactant along the interface during each pulsation cycle.

In contrast, when surfactant is initially present on the core interface, its distribution remains nearly uniform throughout the pulsation cycle, with only slight shifts in the location of maximum accumulation along the lateral sides of the core, as shown in Figure 8d–8e. Consequently, the resulting interfacial tension gradients and Marangoni stresses are significantly weaker than those observed on the shell interface (Figure 9c) and remain of comparable magnitude during both the crest and trough phases. As a result, the tangential interfacial velocity along the core interface (Figure 9d) is approximately one-fourth of that observed when the surfactant is initially distributed over the shell interface (case (C1)), confirming that surfactant confined to the inner interface exerts only a weak influence on the overall hydrodynamics of the compound droplet, both under steady and pulsatile inflows.

Overall, the presence of surfactant retards the droplet, amplifies deformations, significantly influences droplet shape and reduces core eccentricities compared to the clean case, under both steady and pulsatile inflows. The magnitude of these effects depends on the interface on which the surfactant resides.

### Effect of *St* and *Pe*

In this section, the influence of pulsatile forcing on the dynamics of an insoluble surfactant-laden compound droplet is investigated by systematically varying the *St* and*Pe*. The Strouhal number, $St=\frac{\omega }{2\pi }\sqrt{\frac{{\mathrm{D̃}}_{s}}{g}}$ , represents the ratio of the characteristic hydrodynamic response time, $\sqrt{\frac{{\mathrm{D̃}}_{s}}{g}}$ , to the pulsation time scale, 2π/ *ω*. This number was obtained on taking the characteristic velocity scale to be $\sqrt{g{\mathrm{D̃}}_{s}}$ . Accordingly, three values of *St* (0.5, 1 and 2) are considered. A lower Strouhal number corresponds to a smaller angular frequency *ω* and hence a longer pulsation period. At *St* = 0.5, the pulsation period is much larger than the characteristic hydrodynamic response time, and the compound droplet interface immediately responds to the imposed pulsation. Therefore, the droplet interface closely follows the pulsations undergoing strong, high amplitude deformation before the flow reverses (Figure 10a). In contrast, at higher Strouhal numbers (*St* = 2), the pulsation period becomes shorter and within a given time, the droplet experiences more frequent oscillations with a smaller velocity and deformation amplitude than at lower Strouhal numbers. Hence, increasing *St* not only smoothens droplet deformation but also attenuates droplet response and increases its residence time (Figure 10b).

**Figure 10:**
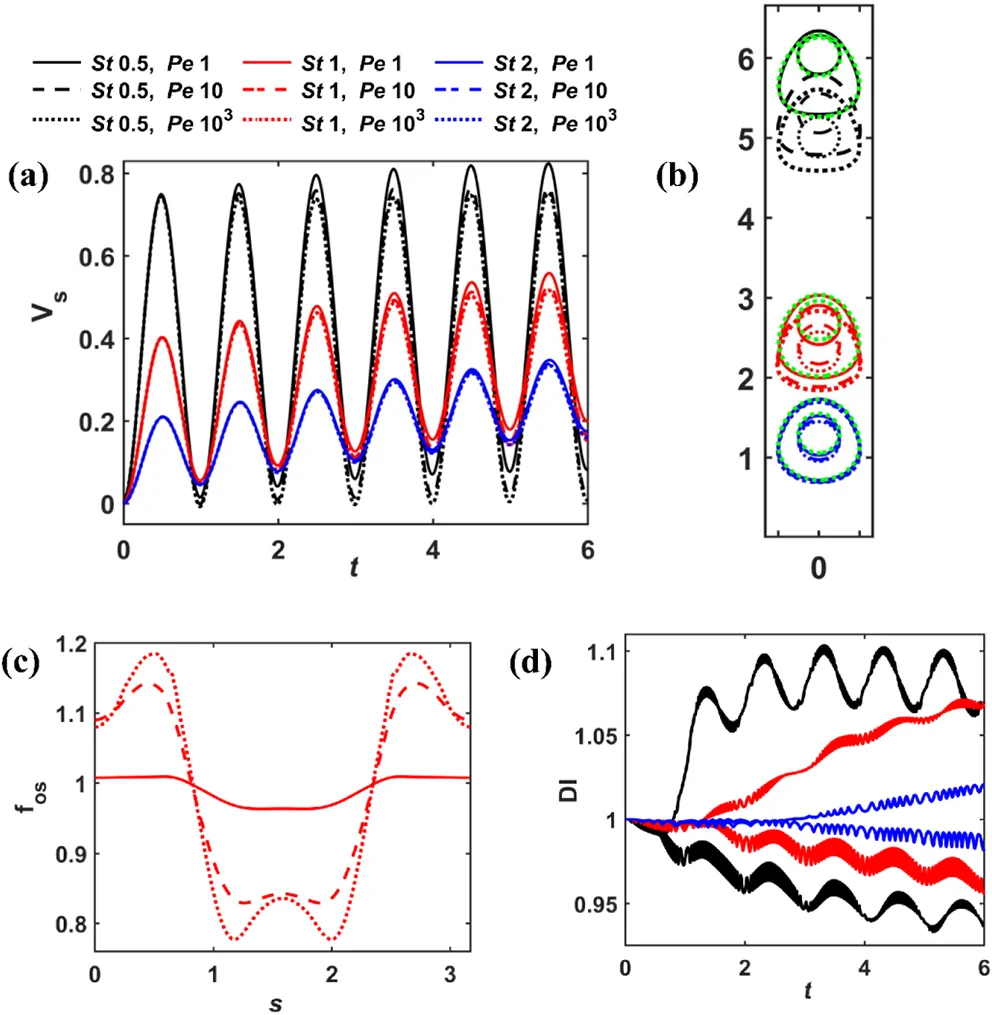
Dynamics of a compound droplet with surfactant present only on the outer shell interface, case (C1). (a) velocity as a function of time for various *St* and *Pe*. (b) Droplet shape and position corresponding to different *St* and *Pe*. The black, blue and red curves, depict compound droplets at *St* = 0.5, 1 and 2, within each set, the solid, dashed and dotted curves represent *Pe* = 1, 10, and 1000 respectively, as clearly indicated in the legend of panel (a). The green dotted drops correspond to a clean interface at *St* = 0.5, 1 and 2 (c) Surfactant concentration vs arc length at *St* = 1, for *Pe* = 1, 10 and 1000. (d) Deformation index vs time for various *St*. The simulation parameters *Ar* = 25, Δ*p* = 0.5, ξ = 0.6, *β* = 0.5, *Bo_os_* = *Bo_sc_* = 5, ρ̂*_o_* =1, ρ̂*_^s_* = 0.8, ρ̂*_c_* = 0.8, μ̂*_o_* = 1, μ̂*_s_* = 0.5, μ̂*_c_* = 0.5, *A* = 1, *R_s_* = 0.75 *R* and *R_c_* = 0.5 *R_s_*.

Similarly, the Péclet number, *Pe* describes the relative importance of convective to diffusive mass transport of surfactant along the interface. For each value of *St*, three *Pe* values: 1, 10 and 1000, are analyzed. A value of 1 represents strong diffusion of surfactant over the interface while *Pe* = 1000 indicates that convective transport dominates. The primary goal of this section is therefore to understand the coupled effects of pulsatile forcing and the surfactant transport, and to determine how pulsation modifies surfactant distribution and consequently affects the hydrodynamics of the compound droplet.

In the present analysis, the surfactant is initially distributed only on the outer shell while the inner core remains clean, corresponding to case (C1). Figure 10 illustrates the droplet dynamics for various combinations of *St* and *Pe*. The black, red and blue curves describe *St* = 0.5, 1 and 2, respectively, while within each *St*, the solid, dashed and dotted curves represent *Pe* = 1, 10, and 1000. As can be seen from Figure 10a, the shell velocity *V_s_* oscillates with a larger amplitude and responds instantaneously to pulsatile forcing at *St* = 0.5 as compared to 2. The droplet exhibits higher average shell velocities and hence travels a longer distance in the channel at *St* = 0.5 compared to *St* = 2.

For all values of *St*, increasing *Pe* from 1 to 10^3^, leads to a marginal drop in the shell velocity.

For each *St*, the position and shape corresponding to different *Pe* are compared with those of a clean droplet, in Figure 10b at *t* = 6. It is observed that for all *St*, increasing Pe from 1 to 1000, shows a significant droplet retardation, with the effect being pronounced at lower *St* (*St* = 0.5) than at *St* = 2. At *Pe* = 1, the dynamics and shape of the surfactant-laden compound droplet closely resemble those of a clean droplet. Higher *Pe* values also result in a less eccentric core, with the core remaining nearly centered within the shell.

Figure 10(c) depicts the surfactant distribution along the arc length at *St* = 1, for various values of *Pe*. The surfactant concentration remains almost uniform at *Pe* = 1, indicating a strong diffusive transport. As *Pe* increases to 10 and subsequently to 10^3^, the surfactant gets advected toward the rear, leading to greater accumulation close to the tail and minimal concentration near the front. This nonuniform distribution generates significant Marangoni stresses, which retard the motion of the compound droplet. These trends are consistent with earlier studies on surfactant-laden simple droplets subjected to steady Poiseuille flow.^19^ Finally, the temporal variation of the Deformation Indices (*DI*) of both shell and core is depicted in Figure 10d for different values of *St*. The shell experiences greater deformation at lower *St*, due to the stronger frequency of oscillation, while the magnitude reduces as *St* is increased to 2. Moreover, *St* = 0.5 displays a higher shell prolation and core oblation compared to *St* = 2. All the above observations indicate that although an increase in *Pe* enhances droplet retardation for a given *St*, variations in *St* exhibit a more significant influence on the overall hydrodynamics than changes in *Pe*.

## Conclusions

In this study, the dynamics of a deformable compound droplet laden with an insoluble surfactant and suspended in an outer continuous fluid, within a confined vertical channel has been investigated using an in-house solver based on the level set method. The analysis systematically considers three different initial surfactant distribution scenarios: (C1) where surfactant is initially present only on the outer shell interface, while the core remains clean, (C2) in which surfactant is solely present on the inner core and (C3) where surfactant is distributed on both interfaces. These configurations are motivated by those reported in previous experimental studies. The results are systematically compared with the behavior of a clean compound droplet. The droplet dynamics under both steady Poiseuille and time-dependent pulsatile inflows are investigated. To the best of the authors’ knowledge, this work represents the first investigation of confined surfactant-laden compound droplets that simultaneously considers multiple surfactant distribution scenarios and different inflow conditions. The key findings of this study are

1 The surfactant concentration gradients (and the Marangoni stresses) are significantly stronger on the outer shell interface, which is directly exposed to the external flow, compared to the inner core interface, which remains encapsulated and relatively isolated from external inflow. Consequently, the presence of surfactant on the shell has a much stronger influence on the overall droplet dynamics than surfactant on the core, under both steady and pulsatile inflows.
2 The presence of surfactants under both steady and pulsatile inflows leads to a noticeable reduction in the compound droplet speed compared to the clean case. The translation velocity follows the order: (C) ∼ (C2) >;(C1) >;(C3). When surfactant is present only on the core interface, the droplet dynamics remain comparable to that of a clean droplet, for various core sizes. In contrast, the presence of surfactants on the shell (cases (C1) and (C3)), significantly alter the droplet speed, deformation and core eccentricity, under both steady and pulsatile flows.
3 Under pulsatile inflows, the deformation indices (*DI*) and core eccentricities (*e*) oscillate about a steady value indicating pronounced time-dependent shape variations and core positions of the compound droplet. The presence of surfactant on the shell/both interfaces results in a greater shell elongation which in turn stretches the inner core to a nearly circular configuration, compared to a clean droplet.
4 For a clean droplet and when surfactant is present only on the core, the core travels toward the front of the shell, under both steady and pulsatile inflows, revealing a greater core eccentricity. However, the presence of surfactants on the shell/both interfaces generates Marangoni stresses which retard the droplet, resulting in the core remaining nearly centered within the shell.
5 *St* has a stronger influence on the droplet dynamics than *Pe*. A lower *Pe* results in droplet behavior similar to that of a clean droplet. Although increasing *Pe* for different *St* values cause a reduction in droplet velocity, this effect is less pronounced than the influence of *St*. Lower *St* values lead to larger droplet deformations, with the droplet adjusting to the outer flow instantaneously and traveling greater distance, in comparison to a larger *St*, where the droplet motion is smoother with reduced deformations.

Overall, the results provide a comprehensive understanding of how the initial surfactant distribution, interfacial surface transport, and unsteady flow conditions influence the motion, deformation, and stability of compound droplets within confined microchannels. The findings from this study not only advance our fundamental understanding of compound droplet dynamics but also provide valuable insights for designing controlled encapsulation systems, emulsion stabilization strategies, and droplet-based microfluidic processes. Although the present model considers flow within a 2D channel, future studies can extend the analysis to incorporate more realistic 3D effects.

## Supplementary Material



## Notes

Additional validation studies, time-step convergence analyses, and an investigation of the effect of core size on the dynamics of the surfactant-laden compound droplet (PDF)
