# Supplementary material for: Hydrodynamics of a Confined Surfactant-Laden Compound Droplet under Steady and Pulsatile Flows
Source: Langmuir. 2026 Sep 23;42(39):28873–91. doi: 10.1021/acs.langmuir.6c02929 (PMC13679093; doi:10.1021/acs.langmuir.6c02929)
Supplement: Supplementary file 1 [file la6c02929_si_001.pdf]

Supporting Information

Hydrodynamics of a Confined Surfactant-Laden Compound Droplet under Steady and Pulsatile Flows

Shubham Lanjewar, Priyesh Tongaria, Sundari Ramji*

Department of Chemical Engineering, BITS Pilani K K Birla Goa Campus, India 403726

* Corresponding author

Email id. sundarir@goa.bits-pilani.ac.in

Table of Contents

Validation Study 1: Surfactant transport in a stationary droplet

Validation Study 2: Surfactant transport in a translating droplet

Time-step convergence study

Effect of the core size on the dynamics of surfactant-laden compound droplet

Validation Study 1: Surfactant transport in a stationary droplet

In this test case, adapted from Xu & Zhao [1], a stationary droplet of unit radius is considered within a square computational domain of [-2,2] x [-2,2]. The droplet is coated with an insoluble surfactant, initially distributed as $f(\theta,0) = \sin(\theta) + 2$. Since the droplet remains stationary, surfactant transport occurs solely through diffusion. Accordingly, the governing equation simplifies to $\frac{\partial f_j}{\partial t} = \nabla_s^2 f_j$. The corresponding analytical solution, also taken from Xu and Zhao, is given by $f(\theta,t) = e^{-t}\sin(\theta) + 2$. The simplified equation is solved numerically, and the time evolution of the surfactant concentration along the droplet interface is plotted against arc length s, as shown in **Fig. S1**. Arc length $\boldsymbol{s}$ is defined as the product of droplet radius and theta [2], where theta is zero (π) at the rear (front) as depicted in **Fig. 1(b)**. Over time (t = 0.5, 1, 2, 6), the concentration profile becomes increasingly uniform, with the peak concentration diminishing—indicating diffusion-driven redistribution toward equilibrium. The numerical results (solid blue lines) show excellent agreement with the analytical solution (dashed red lines), thereby validating the accuracy of the implemented diffusion model.

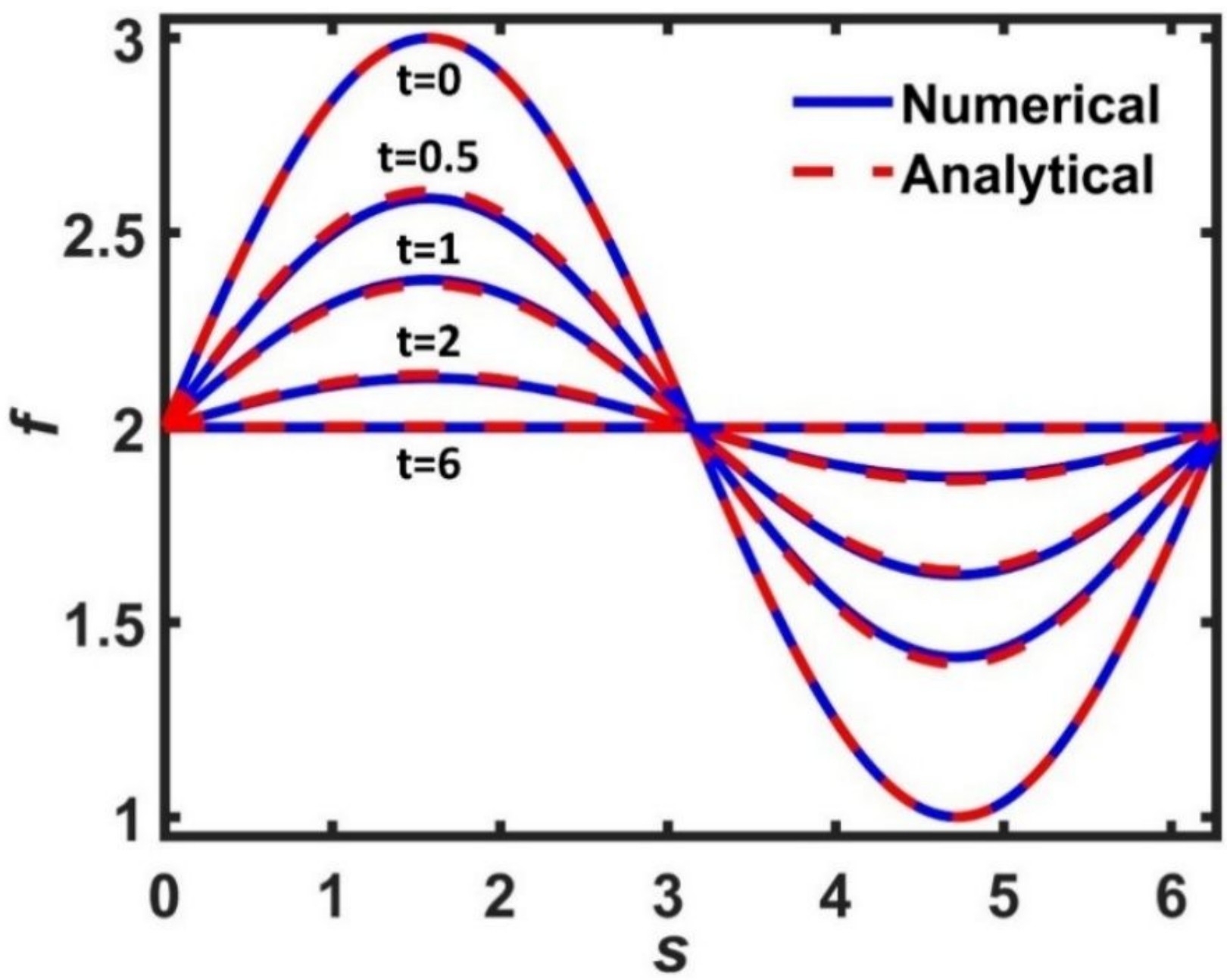

Fig. S1 Comparison of the numerical and analytical solutions of surfactant concentration along the arc length ($\boldsymbol{s}$), for a stationary droplet at various time instances. Grid size: 161 x 161

Validation Study 2: Surfactant transport in a translating droplet

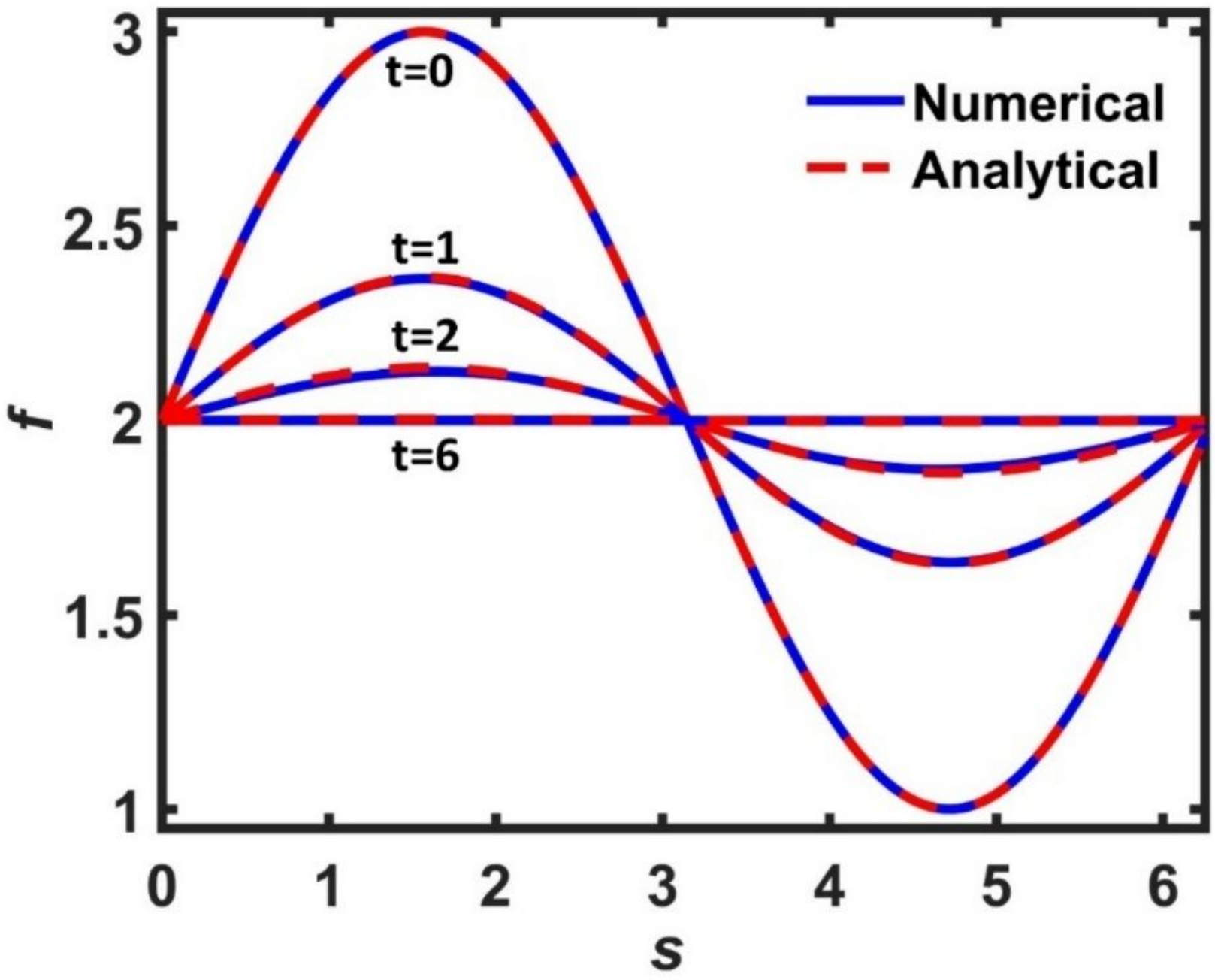

Fig. S2 Surfactant concentration vs. arc length. Comparison of numerical and analytical solutions for a translating droplet at different times. Grid size: 251x101

The second benchmark test, also adopted from Xu & Zhao (2003) [1], comprises of a circular droplet with a radius of 2 units positioned within a periodic domain spanning [-3,5] x [-3,3]. The initial surfactant concentration is given by $f(\theta,0) = \sin(\theta) + 2$. The droplet translates with a uniform, steady dimensionless velocity of magnitude one, introducing both convection and diffusion into the surfactant transport (**Eq. 16**). The corresponding analytical solution is $f(\theta,t) = e^{-\frac{t}{4}}\sin\left(\theta(t)\right) + 2$. **Fig. S2** presents a comparison between the analytical solution (red dashed line) and the numerical results (solid blue line), demonstrating strong agreement and thus validating the accuracy of the full numerical implementation of the surfactant transport equation.

Timestep convergence study

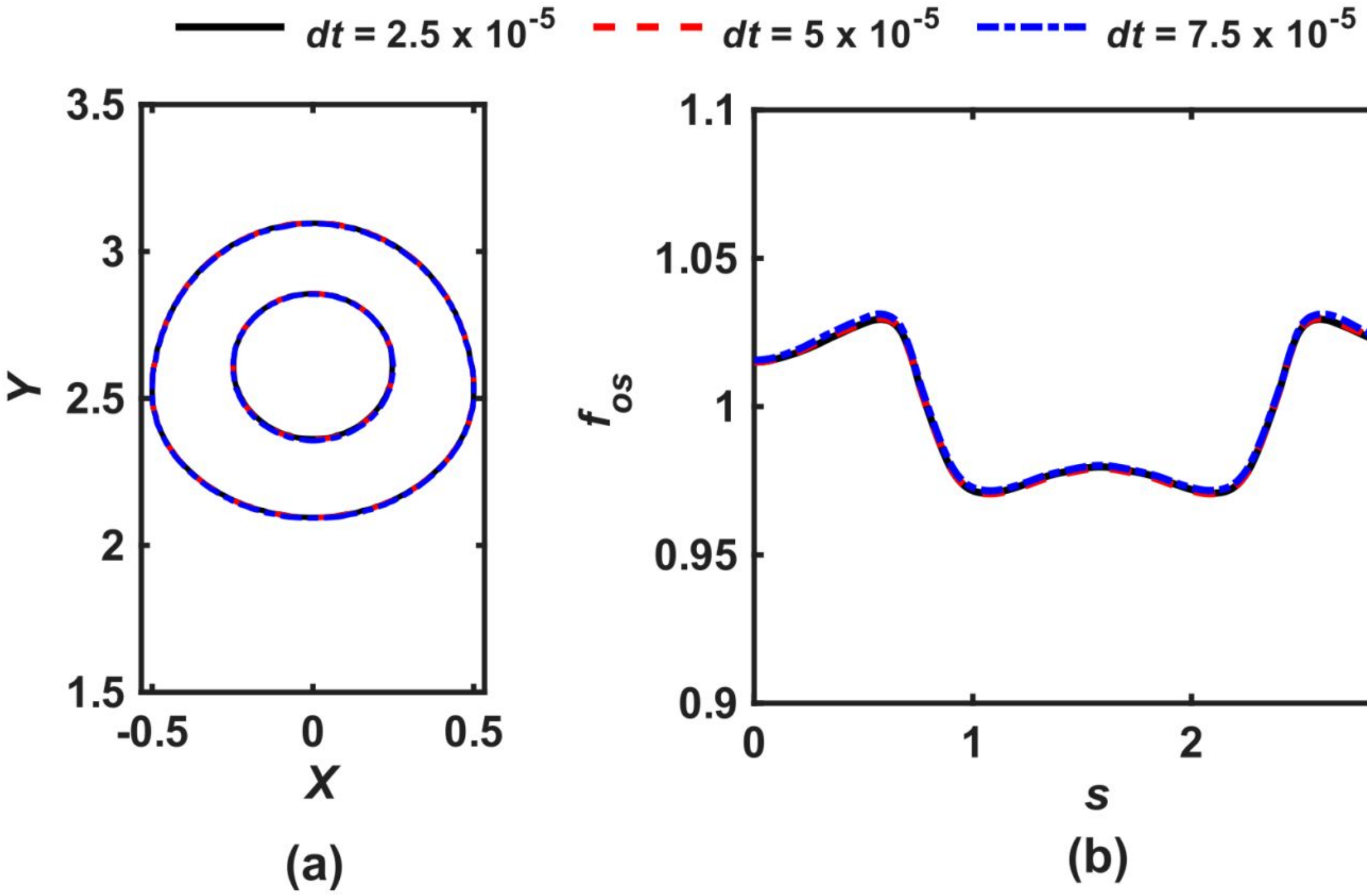

Fig. S3 Timestep convergence study for various dt. **(a)** Droplet position and shape **(b)** surfactant concentration vs. arc length at t = 30. Simulation parameters are $R_c = 0.5\ R_s,\ Bo_{os} = Bo_{sc} = 1$, $Re = 10$, $\hat{\mu}_s = \hat{\mu}_c = 1$, $\hat{\rho}_s = \hat{\rho}_c = 1$, $\Delta p = 0.5$, $\xi = 0.6$, $\beta = 0.5$, $Pe = 10$, St = 1 and A = 0.

A time-step convergence study was performed to determine an appropriate time step, dt, that yields numerical solutions independent of the temporal discretization. Three progressively smaller time steps, dt =7.5 x 10^{-5}, 5 x 10^{-5} and 2.5 x 10^{-5}, were considered while keeping the grid size and all other simulation parameters unchanged. A representative case, namely case (C1) with the parameters specified in the caption of **Fig. S3**, was selected for the study. The droplet position and shape, translational velocities, and surfactant concentration distributions were found to overlap for all three-time steps, demonstrating temporal convergence. The appropriate time step, dt, depended on the grid resolution and the governing flow parameters, including the Strouhal number, Reynolds number, pulsation amplitude, and imposed pressure drop. Accordingly, a suitable value of dt was selected for each simulation to ensure numerical stability and temporal convergence.

Effect of the core size on the dynamics of surfactant-laden compound droplet

To examine whether our conclusions are influenced by the chosen compound droplet geometry, additional simulations were performed comparing clean (C), surfactant on shell (C1) and surfactant on core (C2) by varying the core to shell radius ratio over a physically relevant range, $R_{C-S} = \frac{R_C}{R_S} =$ 0.4, 0.5, 0.6 and 0.7. To isolate the influence of surfactant distribution, the densities and viscosities of all three phases were kept identical, thereby eliminating the effects of density and viscosity contrasts. The droplet positions and their corresponding translation velocities were quantified for all cases are shown in **Fig. S3**.

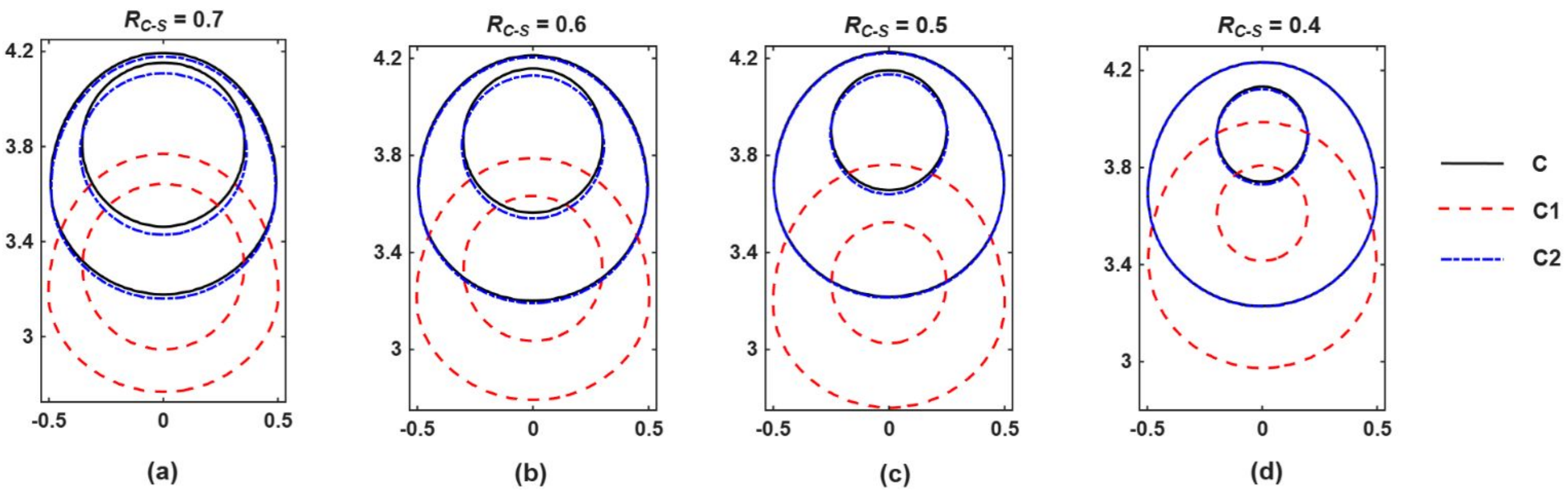

Fig. S3 Influence of core size holdup on the dynamics of a compound droplet. The position and shape of the compound droplet are plotted for various core sizes, $R_{C-S} = 0.7, 0.6, 0.5$ and 0.4 in **(a)**, **(b)**, **(c)** and **(d)** respectively. Within each subplot, the droplet position for clean (C), surfactant present on shell (C1) and surfactant present on core (C2) are compared. Other simulation parameters are $Ar = 10,\ Pe = 10,\ \Delta p = 0.5, \xi = 0.6,\ \beta = 0.5, Bo_{os} = Bo_{sc} = 1, \hat{\rho}_o = \hat{\rho}_s = \hat{\rho}_c = 1, \hat{\mu}_o = \hat{\mu}_s = \hat{\mu}_c = 1, R_s = 0.925R$.

As evident from **Fig. S3**, for all core sizes considered, the clean droplet and the droplet with surfactant confined to the core interface (C2) translate at nearly identical velocities, whereas the presence of surfactant on the shell interface (C1) results in a noticeably lower droplet velocity. Although the influence of the surfactant for case (C2) increases slightly with increasing core size, it remains significantly weaker than case (C1) throughout the entire range investigated. Specifically, the relative velocity difference between cases (C2) and (C) decreases from approximately 0.6% at $R_{C-S} = 0.7$ to 0.2% at $R_{C-S} = 0.4$. In contrast, the corresponding difference between cases C1 and C ranges from approximately 15% to 4.5% over the same range of core sizes. This confirms that surfactant located on the shell interface has a substantially greater influence on the dynamics of a compound droplet than surfactant confined to the core interface

References

(1) Xu, J.-J.; Zhao, H.-K. An Eulerian Formulation for Solving Partial Differential Equations Along a Moving Interface. *J. Sci. Comput.* **2003**, *19* (1), 573–594. https://doi.org/10.1023/A:1025336916176

(2) Xu, J. J.; Shi, W.; Lai, M. C. A Level-Set Method for Two-Phase Flows with Soluble Surfactant. *J. Comput. Phys.* **2018**, *353*, 336–355. https://doi.org/10.1016/j.jcp.2017.10.019
